# Transcending the challenge of evolving resistance mechanisms in *Pseudomonas aeruginosa* through β-lactam-enhancer-mechanism-based cefepime/zidebactam

**DOI:** 10.1128/mbio.01118-23

**Published:** 2023-10-27

**Authors:** Andrea M. Hujer, Steven H. Marshall, Andrew R. Mack, Kristine M. Hujer, Yamuna Devi Bakthavatchalam, Kushal Umarkar, Snehal R. Palwe, Swapna Takalkar, Prashant R. Joshi, Rahul Shrivastava, Hariharan Periasamy, Sachin S. Bhagwat, Mahesh V. Patel, Balaji Veeraraghavan, Robert A. Bonomo

**Affiliations:** 1 Research Service, Louis Stokes Cleveland Department of Veterans Affairs, Cleveland, Ohio, USA; 2 Department of Medicine, Case Western Reserve University School of Medicine, Cleveland, Ohio, USA; 3 Department of Molecular Biology and Microbiology, Case Western Reserve University School of Medicine, Cleveland, Ohio, USA; 4 Department of Clinical Microbiology, Christian Medical College, Vellore, Tamil Nadu, India; 5 Wockhardt Research Centre, Aurangabad, Maharashtra, India; 6 Departments of Pharmacology, Biochemistry, and Proteomics and Bioinformatics, Case Western Reserve University School of Medicine, and the CWRU-Cleveland VAMC Center for Antimicrobial Resistance and Epidemiology (Case VA CARES), Cleveland, Ohio, USA; MedImmune, Gaithersburg, Maryland, USA

**Keywords:** ß-lactamases, antibiotic resistance, *Pseudomonas aeruginosa*, cefepime, zidebactam, ß-lactam enhancer, ß-lactamase inhibitor

## Abstract

Multi-drug resistant (MDR) *Pseudomonas aeruginosa* harbor a complex array of β-lactamases and non-enzymatic resistance mechanisms. In this study, the activity of a β-lactam/β-lactam-enhancer, cefepime/zidebactam, and novel β-lactam/β-lactamase inhibitor combinations was determined against an MDR phenotype-enriched, challenge panel of *P. aeruginosa* (*n* = 108). Isolates were multi-clonal as they belonged to at least 29 distinct sequence types (STs) and harbored metallo-β-lactamases, serine β-lactamases, penicillin binding protein (PBP) mutations, and other non-enzymatic resistance mechanisms. Ceftazidime/avibactam, ceftolozane/tazobactam, imipenem/relebactam, and cefepime/taniborbactam demonstrated MIC_90_s of >128 mg/L, while cefepime/zidebactam MIC_90_ was 16 mg/L. In a neutropenic-murine lung infection model, a cefepime/zidebactam human epithelial-lining fluid-simulated regimen achieved or exceeded a translational end point of 1−log_10_ kill for the isolates with elevated cefepime/zidebactam MICs (16–32 mg/L), harboring VIM-2 or KPC-2 and alterations in PBP2 and PBP3. In the same model, to assess the impact of zidebactam on the pharmacodynamic (PD) requirement of cefepime, dose-fractionation studies were undertaken employing cefepime-susceptible *P. aeruginosa* isolates. Administered alone, cefepime required 47%–68% *f*T >MIC for stasis to ~1 log_10_ kill effect, while cefepime in the presence of zidebactam required just 8%–16% for >2 log_10_ kill effect, thus, providing the pharmacokinetic/PD basis for in vivo efficacy of cefepime/zidebactam against isolates with MICs up to 32 mg/L. Unlike β-lactam/β-lactamase inhibitors, β-lactam enhancer mechanism-based cefepime/zidebactam shows a potential to transcend the challenge of ever-evolving resistance mechanisms by targeting multiple PBPs and overcoming diverse β-lactamases including carbapenemases in *P. aeruginosa*.

**IMPORTANCE:**

Compared to other genera of Gram-negative pathogens, *Pseudomonas* is adept in acquiring complex non-enzymatic and enzymatic resistance mechanisms thus remaining a challenge to even novel antibiotics including recently developed β-lactam and β-lactamase inhibitor combinations. This study shows that the novel β-lactam enhancer approach enables cefepime/zidebactam to overcome both non-enzymatic and enzymatic resistance mechanisms associated with a challenging panel of *P. aeruginosa*. This study highlights that the β-lactam enhancer mechanism is a promising alternative to the conventional β-lactam/β-lactamase inhibitor approach in combating ever-evolving MDR *P. aeruginosa*.

## INTRODUCTION

*Pseudomonas aeruginosa* infections are often difficult to treat particularly in patients admitted to intensive care units with immunosuppression and other comorbidities. Often these patients have received inappropriate empiric therapy or are infected with multi-drug (MDR) or extreme-drug resistant (XDR) pathogens. The successful proliferation of high-risk MDR/XDR clones of *P. aeruginosa* is a consequence of the organism’s ability to manifest intrinsic and acquired resistance mechanisms, in turn, challenging the approaches traditionally employed for the discovery of novel antibiotics (1, 2).

Despite the introduction of several anti-pseudomonal antibiotics in the past decade, the conundrum of resistance mechanisms composed of hyper-efflux, impermeability, and β-lactamases in *P. aeruginosa* continues to pose therapeutic uncertainty during every treatment episode. Older anti-pseudomonal drugs (ceftazidime, cefepime, and piperacillin/tazobactam) are compromised by the hyper-production of pseudomonal-derived cephalosporinases (PDCs) (3), while OprD inactivation and/or hyper-efflux mechanisms impact carbapenems (4). Target modifications in the background of efflux compromise the activity of fluoroquinolones and aminoglycosides (5). As a result, in the United States, currently, 20%–30% of *P. aeruginosa* isolates display an MDR phenotype, which prompted the Centers for Disease Control and Prevention (CDC) to designate this pathogen as a “serious” threat. Likewise, the World Health Organization (WHO) designates *P. aeruginosa* as a “critical” pathogen for which new antibiotics are urgently needed (6–8). A recent CDC report describes a disturbing trend of a 32% rise in hospital-onset infections caused by MDR *P. aeruginosa* in 2020 highlighting the “collateral damage” of the COVID-19 pandemic (9).

Of late, much of the antibiotic discovery efforts have been directed towards finding novel β-lactam or β-lactamase inhibitor-based combinations to overcome diverse β-lactam-impacting resistance mechanisms in Gram-negatives including carbapenem-resistant-Enterobacterales, -*P. aeruginosa* and -*Acinetobacter baumannii*. Such efforts have led to the development of combinations such as ceftazidime/avibactam, ceftolozane/tazobactam, imipenem/relebactam, and cefepime/taniborbactam that show improved anti-pseudomonal activity compared to older therapies. However, a “coverage gap” continues to exist, as despite the chemical diversity of newer β-lactamase inhibitors, many are unable to inhibit the entire range of clinically significant β-lactamases in this pathogen (10). Also, reports of newer PDC variants continue to challenge the inhibitory activity of novel inhibitors paired with cephalosporins (11–13).

Recently, an unconventional discovery approach based on novel β-lactam enhancer action has been reported for phase 3-stage cefepime/zidebactam (WCK 5222) (14). The enhancer action of this combination is mediated by zidebactam, a novel bicyclo-acyl hydrazide (derived from diazabicyclooctane) possessing a potent penicillin-binding protein (PBP) 2 binding activity. When zidebactam is combined with PBP3-targeting cefepime, a mechanistic synergy is triggered resulting in the enhancement of cefepime’s bactericidal activity, both *in vitro* and *in vivo* against a broad spectrum of Gram-negative pathogens expressing diverse carbapenem-impacting resistance mechanisms (15, 16). Against *A. baumannii,* pharmacodynamic (PD) studies have established that zidebactam lowers cefepime’s exposure required for *in vivo* bactericidal activity (17) which forms the basis for the combination’s efficacy against isolates with cefepime/zidebactam minimum inhibitory concentrations (MICs) of up to 64 mg/L in translational animal infection models (18, 19).

For challenging *P. aeruginosa* infections involving MDR/XDR isolates, the potential clinical utility of novel agents developed through the aforementioned approaches would rely on their ability to overcome a multiplicity of resistance mechanisms. To investigate this aspect, a set of 108 whole genome sequenced heterogeneous *P. aeruginosa* isolates collected from the U.S. harboring diverse β-lactam-impacting resistance mechanisms was assembled. The *in vitro* activity of cefepime/zidebactam and novel anti-pseudomonal β-lactam/β-lactamase inhibitor combinations was determined against this panel. Furthermore, isolates with cefepime/zidebactam MICs > 8 mg/L (higher than the cefepime susceptible breakpoint) were employed in a translational neutropenic murine lung infection study to assess the *in vivo* efficacy of a human epithelial-lining fluid-simulated regimen (ELF-HSR) of cefepime/zidebactam. Finally, using the same model, the pharmacokinetic/pharmacodynamic (PK/PD) basis of *in vivo* efficacy of cefepime/zidebactam against *P. aeruginosa* was deciphered by studying the impact of zidebactam on cefepime’s % *f*T >MIC requirement. For this purpose, cefepime-susceptible *P. aeruginosa* were used, as such isolates enable identifying the standalone cefepime’s % *f*T >MIC requirement which then can be compared with cefepime’s requirement in the presence of zidebactam.

## RESULTS

### Genetic composition of challenge isolates

**Fig 1:**
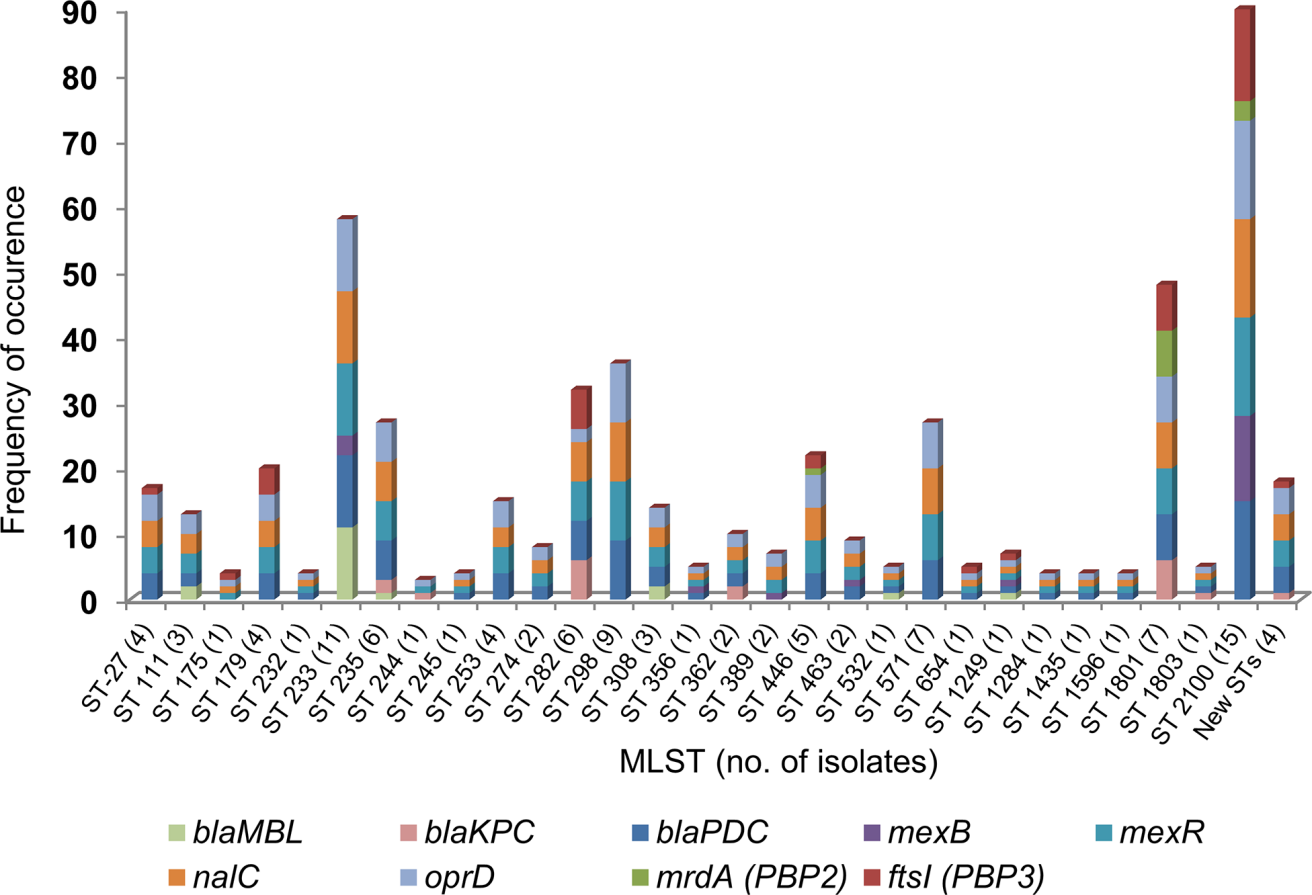
Diversity and frequency in occurrence of resistance mechanisms (β-lactamases and mutations in proteins) in *P. aeruginosa* elucidated by whole genome sequencing (*n* = 108). *bla*_MBL_: 17 were VIM-2 and one was NDM-1, *bla*_KPC_: 18 were KPC-2 and one was KPC-5.

Analysis of the whole genome sequences revealed that the study isolates (*n* = 108) belonged to a diverse genetic background with at least 29 distinct, previously reported sequence types (STs). When analyzed in comparison to the genome of the reference isolate (*P. aeruginosa* PAO1), the study isolates demonstrated many nucleotide changes in the genes encoding several key functional proteins (PBP2, PBP3, PDC, AmpR, MexR, MexB, NalC, and OprD) known to be associated with β-lactam resistance in *P. aeruginosa* (Fig. 1; Table S1).

A total of 37 isolates (34.3%) were found to express carbapenemases (KPC, 19 [KPC-2, 18; KPC-5, 1]; MBL, 18 [VIM-2, 17; NDM-1, 1]). Out of 19 KPC-producing *P. aeruginosa*, 6 and 15 isolates were ceftazidime/avibactam (MICs > 8 mg/L) and imipenem/relebactam-non-susceptible (MICs > 2 mg/L), respectively. Among them, one isolate that produced KPC-5 (a KPC variant reported to possess increased hydrolytic activity against ceftazidime) (20) and harbored *oprD* mutations showed resistance to both ceftazidime/avibactam and imipenem/relebactam.

Three of the ceftazidime/avibactam-non-susceptible isolates belonged to ST 1801 and showed simultaneous substitutions in PBP3 (F533L) (21) and PBP2 (A174V, V517M) (22), while the other three (ST 235 and ST 244) isolates had substitutions in proteins involved in efflux regulation (e.g., the V126E substitution in MexR).

Among 15 imipenem/relebactam-non-susceptible isolates, 12 isolates had PBP3 substitutions (either F533L [*n* = 6] or T91A [*n* = 6]). Our analysis showed that F533L substitution in PBP3 was invariably associated with PBP2 substitutions (A174V, V517M).

The V126E substitution in MexR is frequently found in MDR *P. aeruginosa* isolates and has been associated with an increase in the MICs of imipenem in the background of impermeability (23–25). Thus, among KPC-producing isolates, the main reason for ceftazidime/avibactam and imipenem/relebactam non-susceptibilities seems to be linked with changes in PBP3 and efflux in the background of impermeability (OprD mutations). Though the OprD mutations were also observed in the ceftazidime/avibactam and imipenem/relebactam-susceptible isolates, the absence of high-level resistance in the majority of those isolates suggests that the major contributors in raising their MICs are PBP3 modifications and efflux.

Five out of nineteen KPC-producing isolates showed cefepime/zidebactam MICs of 16–32 mg/L (above cefepime’s susceptible breakpoint); they harbored mutations in PBP3 (F533L), PBP2 (A174V, V517M), and NalC (G71E, S209R). In contrast, 16/19 KPC-producing isolates showed cefepime/taniborbactam MICs in the range of 16–128 mg/L probably due to substitutions in multiple proteins including PBP3, OprD, and those related to efflux.

Among non-carbapenemase producing isolates (*n* = 71), in general, the mutations responsible for raising the MICs of different antibiotics were associated with both enzymatic (PDC) and non-enzymatic (efflux and target) mechanisms. Specific mutations that correlated with the increase in both the ceftazidime/avibactam and ceftolozane/tazobactam MICs were more often detected in PBP3 (R504C, F533L) (21, 26) and PDC (E219K, SANC numbering) (27). The non-susceptibility to imipenem/relebactam in non-carbapenemase producers seems to be multifactorial as mutations were observed in PBP3, efflux-related proteins, and OprD. Cefepime/zidebactam MICs of 16–32 mg/L were observed in isolates belonging to ST 356 (*n* = 1), ST 1801 (*n* = 1), and ST 2100 (*n* = 5). In this instance, the major detected mutations were in PBP3 (G63D, R504C, and F533L), PBP2 (A174V, V517M, and G591S), and PDC-537 (P153L, E219K, SANC numbering) (28).

### Comparative *in vitro* activity

Table 1 shows the MIC distribution of antibiotics for the isolates categorized as MBL- or KPC-producers or non-carbapenemase producers. Fig. S2 shows the MICs of cefepime/zidebactam versus other β-lactam/β-lactamase inhibitor combinations for each carbapenemase-producing isolate.

**TABLE 1:** MIC distributions of cefepime/zidebactam and other antibiotics for major resistance groups^*a*^

Organism group	Number of *P. aeruginosa* isolates with indicated MIC (mg/L)									
	≤ 0.25	0.5	1	2	4	8	16	32	64	≥128
KPC producers (*n* = 19)										
Cefepime										19
Cefepime/zidebactam					6	8	2	3		
Cefepime/taniborbactam					1	2	7	1		8
Ceftolozane/tazobactam						3	1	4	5	6
Ceftazidime/avibactam			**1**	**4**	**2**	**6**	3	2	1	
Aztreonam/avibactam						6		9	3	1
Imipenem/relebactam		**1**	**1**	**2**	2	3	3	2	1	4
Meropenem							1	2		16
Meropenem/vaborbactam						2	1	2	3	11
MBL producers (*n* = 18)										
Cefepime							2	3	3	10
Cefepime/zidebactam				3	7	5	2	1		
Cefepime/taniborbactam					2	3	1		6	6
Ceftolozane/tazobactam										18
Ceftazidime/avibactam								4	5	9
Aztreonam/avibactam	1				1	6	3	2	4	1
Imipenem/relebactam			**1**					1	4	12
Meropenem						2	3	1	5	7
Meropenem/vaborbactam					1	2	4	5	4	2
Non-carbapenemase producers (*n* = 71)										
Cefepime			**2**	**2**	**10**	**14**	10	11	12	10
Cefepime/zidebactam		1	1	14	23	25	4	3		
Cefepime/taniborbactam			3	6	15	15	11	6	4	11
Ceftolozane/tazobactam	**4**	**14**	**9**	**8**	**7**	5	3	1	7	13
Ceftazidime/avibactam		**1**	**5**	**7**	**8**	**14**	11	2	7	16
Aztreonam/avibactam					5	6	8	22	15	15
Imipenem/relebactam	**4**	**2**	**13**	**25**	18	8			1	
Meropenem		**2**		**4**	10	11	18	16	7	3
Meropenem/vaborbactam	1	3		4	10	13	25	9	4	2

^*a*^ Susceptible range (FDA criteria) for each agent except for cefepime/zidebactam, cefepime/taniborbactam, aztreonam/avibactam and meropenem/vaborbactam is depicted by boldfaced numbers; for these approved antibiotics, FDA breakpoints are consistent with CLSI breakpoints. MIC of cefepime/zidebactam was determined at 1:1 ratio. A fixed 4 mg/L of inhibitor concentration was used for cefepime/taniborbactam, ceftolozane/tazobactam, ceftazidime/avibactam, and imipenem/relebactam. A fixed 8 mg/L of inhibitor concentration was used for meropenem/vaborbactam.

**Fig 2:**
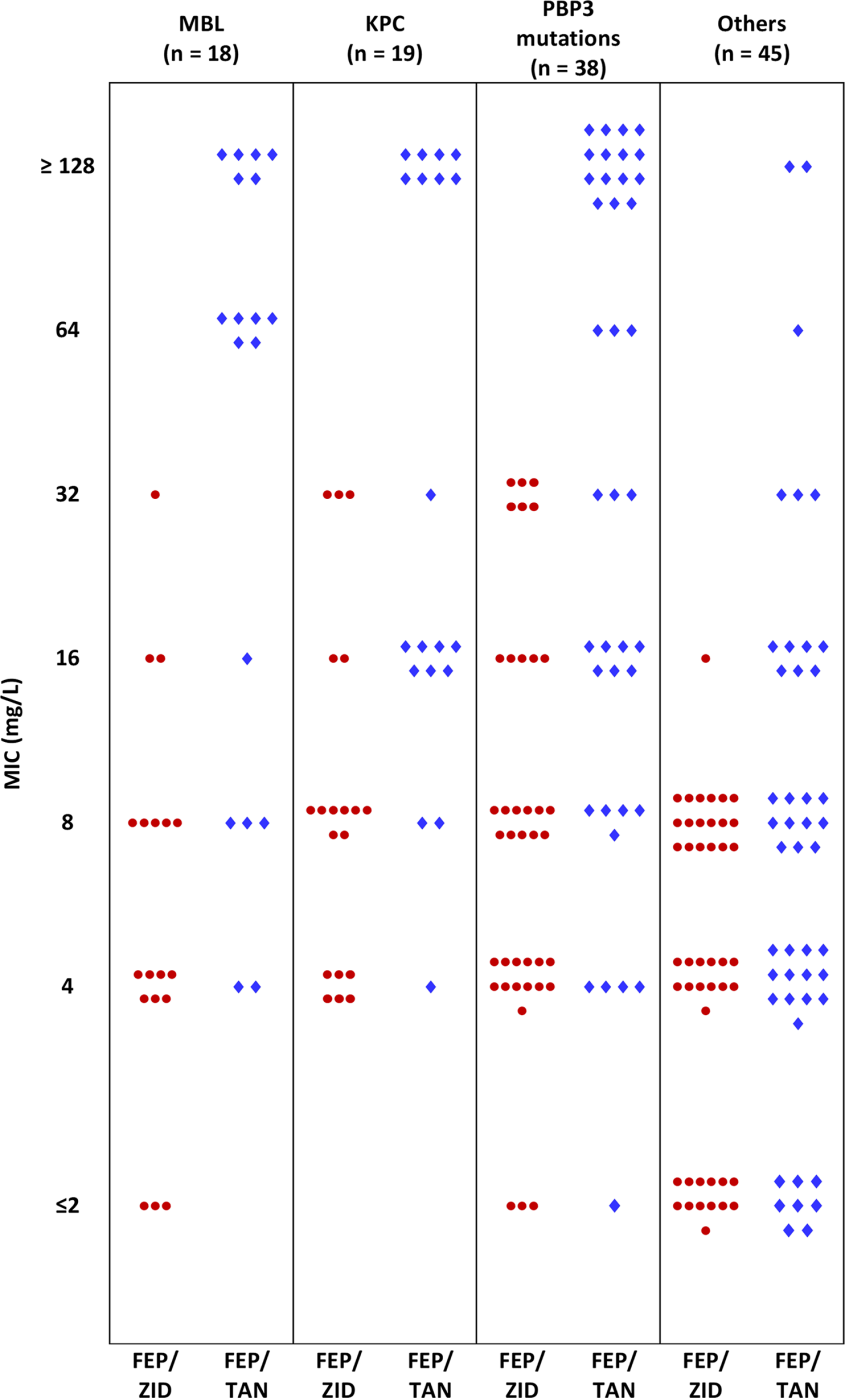
Comparative distribution of MICs of two cefepime-based combinations categorized as per major resistance mechanisms identified through whole genome sequencing of 108 *P. aeruginosa*. Each symbol represents one isolate. FEP/ZID: cefepime/zidebactam; FEP/TAN: cefepime/taniborbactam.

As anticipated, the MBL subset (*n* = 18) was resistant to ceftolozane/tazobactam (18/18), ceftazidime/avibactam (18/18), and imipenem/relebactam (17/18) using FDA breakpoints. Further, adding the MBL inhibitor, taniborbactam (4 mg/L) to cefepime, did not significantly improve the susceptibility to cefepime. While cefepime MICs were 16 to ≥128 mg/L for all MBL isolates, taniborbactam reduced the cefepime MICs to ≤8 mg/L for only 5 of 18 isolates (27.8%) with one additional isolate inhibited at 16 mg/L. For this MBL subset, cefepime/zidebactam was distinctly more active than cefepime/taniborbactam with 15 of 18 isolates (83.3%) inhibited at ≤8 mg/L and all except one were inhibited at ≤16 mg/L (94.4% inhibition) (Fig. 2). Despite the known stability of aztreonam towards MBL hydrolysis, aztreonam/avibactam inhibited just 33.3% of MBL isolates at 8 mg/L.

As stated above, *bla*_KPC_ was found in 19 isolates with KPC-2 in 18 isolates and KPC-5 in the remaining one isolate. All were non-susceptible to ceftolozane/tazobactam. Against this subset, despite avibactam, relebactam, and taniborbactam being known to inhibit KPC, their respective combinations showed limited activity at their corresponding susceptible breakpoints. On the other hand, cefepime/zidebactam MICs ranged from 4 to 32 mg/L; 16/19 were inhibited at ≤16 mg/L (Table 1).

No carbapenemase was detected in the remaining 71 isolates. Regardless, 91.5% of them were non-susceptible to meropenem which indicates enrichment of carbapenem-impacting non-enzymatic resistance mechanisms in this panel. Unexpectedly, 29/71 (40.8%) and 36/71 (50.7%) were non-susceptible to ceftolozane/tazobactam and ceftazidime/avibactam, respectively (Table 1). As described earlier, this non-susceptible population was enriched with substitutions in PBP3 and efflux proteins as well as in PDCs reported to be linked with a rise in ceftolozane/tazobactam and ceftazidime/avibactam MICs (Table S1). Imipenem/relebactam and cefepime/taniborbactam also showed sub-optimal activity; 27/71 (38%) and 32/71 (45.1%) of isolates were non-susceptible to these combinations, respectively.

With regards to non-β-lactam antibiotics, among the entire population, extreme resistance to ciprofloxacin, substantial resistance to amikacin, and potent activity of colistin were observed. The antibiotic panel was also inclusive of meropenem/vaborbactam but not discussed above as, predictably, the addition of vaborbactam did not improve the activity of meropenem (Table 2).

**TABLE 2:** MIC range, MIC_50_ and MIC_90_ of cefepime/zidebactam and other antibiotics for all *P. aeruginosa* (*n* = 108)

Antibiotic/combinations	MIC (mg/L)			% Susceptibility^*a*^
	MIC_50_	MIC_90_	Range	
Cefepime	32	≥128	1 - ≥ 128	25.9
Cefepime/zidebactam	4	16	0.5–32	86.1^*b*^ / 100^*c*^
Cefepime/taniborbactam	16	≥128	1 - ≥ 128	43.5^*b*^
Ceftolozane/tazobactam	16	≥128	0.12 - ≥ 128	38.9
Ceftazidime/avibactam	16	≥128	0.5 - ≥ 128	44.4
Aztreonam/avibactam	32	≥128	0.25 - ≥ 128	23.1^*d*^
Imipenem	32	≥128	0.12 - ≥ 128	9.3
Imipenem/relebactam	4	≥128	0.12 ≥ 128	45.4
Meropenem	32	≥128	0.5 - ≥ 128	5.6
Meropenem/vaborbactam	16	≥128	0.25 - ≥ 128	33.3^*e*^
Ciprofloxacin	16	≥128	0.06 - ≥ 128	12
Amikacin	16	≥128	0.25 - ≥ 128	56.5
Colistin	≤0.25	1	0.03 - ≥ 128	94.4^*f*^

^*a*^ Susceptibility interpreted against FDA criteria.

^*b*^ As per cefepime susceptible breakpoint.

^*c*^ As per cefepime/zidebactam’s proposed PK/PD breakpoint of ≤32 mg/L.

^*d*^ As per aztreonam standalone susceptibility breakpoint.

^*e*^ Based on meropenem susceptible breakpoint of ≤2 mg/L.

^*f*^ Based on CLSI intermediate breakpoint of ≤2 mg/L.

### *In vivo* efficacy

#### Assessment of cefepime/zidebactam efficacy employing ELF-human-simulated regimen

**Fig 3:**
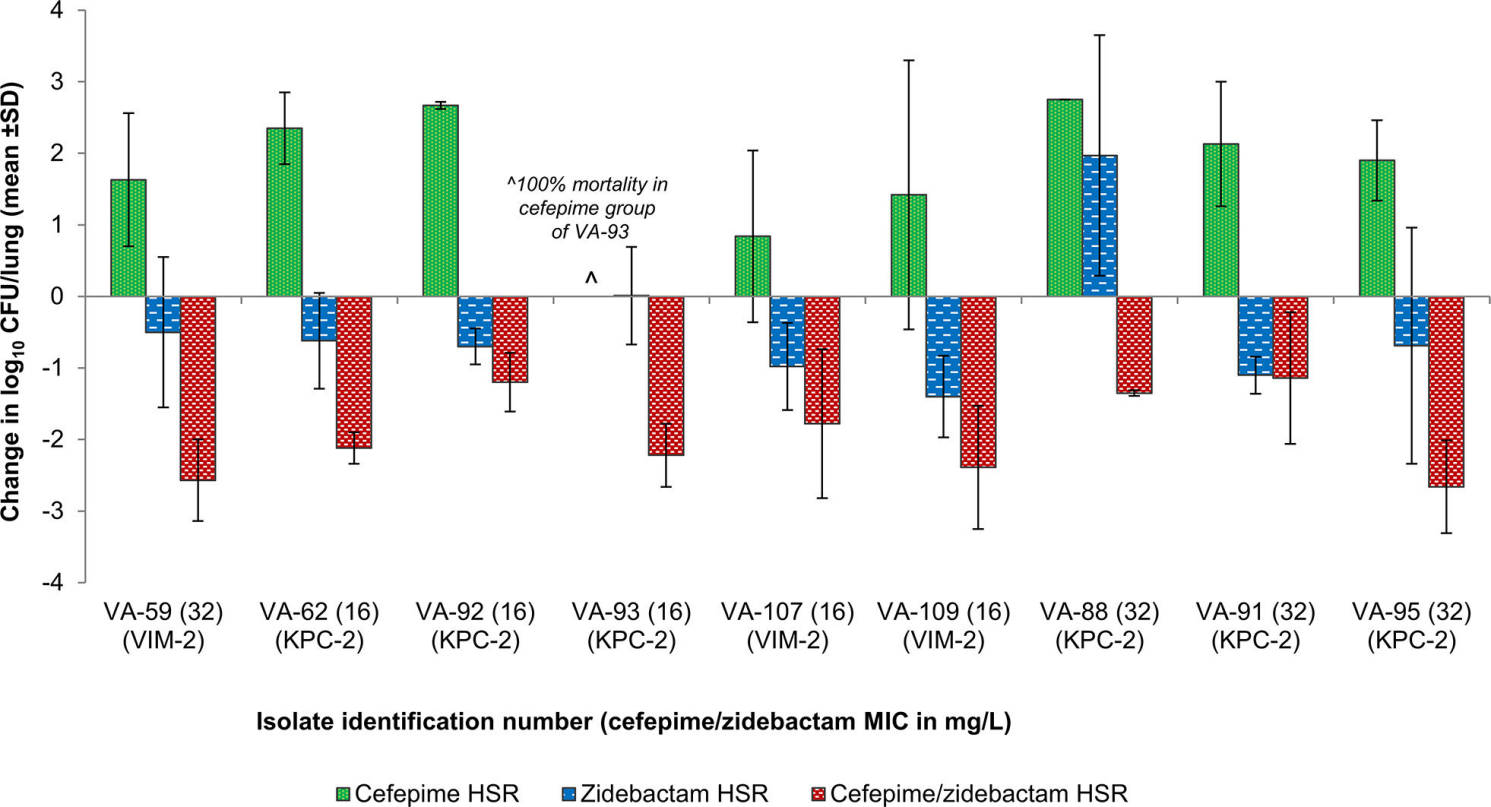
*In vivo* efficacy of human-simulated regimen (HSR) of cefepime/zidebactam in neutropenic murine lung infection model against *P. aeruginosa* with cefepime/zidebactam MICs > 8 mg/L.

For the *in vivo* efficacy study, all the isolates with elevated cefepime/zidebactam MICs (>8 mg/L, *n* = 15) were chosen. However, only 9/15 isolates were able to successfully infect and grow in the lungs of neutropenic mice (the others were unfit) and were included in the efficacy assessment study (Table 3). The bacterial load in the lungs at 0 h ranged from 5.4 to 6.2 log_10_ CFU (mean 5.8 ± 0.2 log_10_ CFU). In the untreated groups, all the mice succumbed to infection by 24 h. Cefepime HSR was not efficacious; a net-growth of >1 log_10_ CFU/lung was noted in 7/9 isolates and in the remaining two isolates, 0.84 log_10_ net growth in VA107 and 100% mortality in VA93 were observed. Zidebactam HSR showed a bactericidal efficacy with a mean net-drop of 0.75 ± 0.42 log_10_ CFU/lung in 8/9 isolates and in a lone isolate, a net-growth of 1.97 log_10_ CFU/lung was observed. In contrast, the cefepime/zidebactam HSR demonstrated pronounced killing in all the studied isolates with a magnitude ranging from 1.1 log_10_ CFU/lung to 2.7 log_10_ CFU/lung (mean 1.9 ± 0.6 log_10_ CFU/lung), thus, exceeding the translational end point of 1-log_10_ kill (Fig. 3).

**TABLE 3:** Major resistance mechanisms identified in *P. aeruginosa* isolates utilized in cefepime/zidebactam *in vivo* efficacy assessment study (cefepime/zidebactam MICs > 8 mg/L)

Isolates^*a*^	MLST	FEP/ZID MICs mg/L	Major resistance mechanisms
VA59	233	32	VIM-2, OXA-4, OXA-486, PDC-3
VA62	1801	16	OXA-486, OXA-10, PDC-3; PBP2 (V517M A174V); PBP3 (F533L)
VA88	1801	32	KPC-2, OXA-10, OXA-486, PDC-3; PBP2 (A174V V517M); PBP3 (F533L)
VA91	1801	32	KPC-2, OXA-10, OXA-486, PDC-3; PBP2 (A174V V517M); PBP3 (F533L)
VA92	1801	16	KPC-2, OXA-10, OXA-486, PDC-3; PBP2 (A174V V517M); PBP3 (F533L)
VA93	1801	16	KPC-2, OXA-10, OXA-486, PDC-3; PBP2 (A174V V517M); PBP3 (F533L)
VA95	1801	32	KPC-2, OXA-10, OXA-486, PDC-3; PBP2 (A174V V517M); PBP3 (F533L)
VA107	233	16	VIM-2, OXA-4, OXA-486, PDC-3
VA109	233	16	VIM-2, OXA-4, OXA-486, PDC-3

^*a*^ The isolates were resistant to other BL/BLI combinations (imipenem/relebactam and ceftolozane/tazobactam) FEP/ZID: cefepime/zidebactam.

### Effect on cefepime’s % *f*T >MIC requirement in the presence of zidebactam

**Fig 4:**
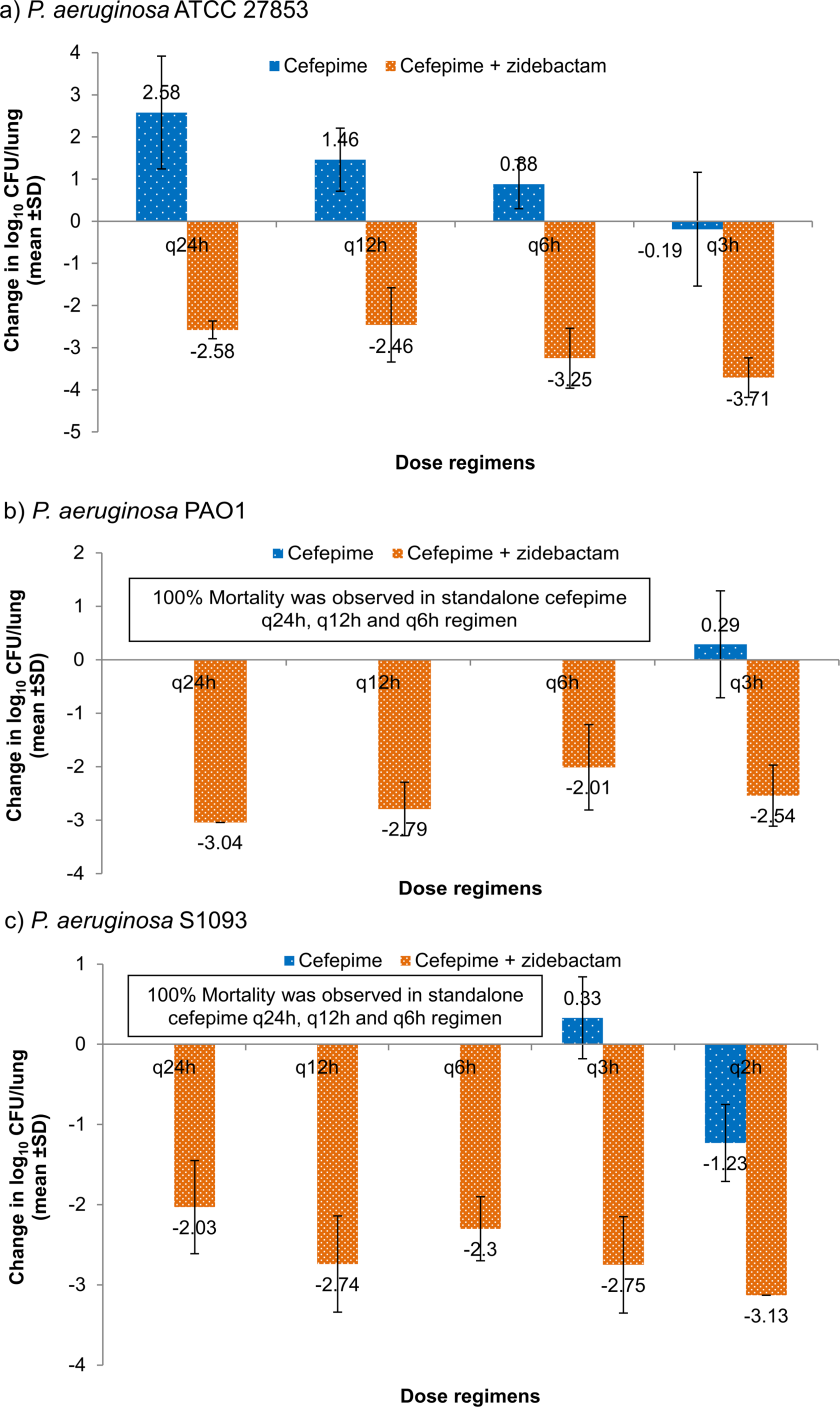
Efficacy of cefepime as standalone and in the presence of zidebactam against cefepime-susceptible *P. aeruginosa* in neutropenic murine lung infection model. Cefepime total dose of 150 mg/kg was administered as single dose (q24h) and in various fractionated regimens [every 12 h (q12h), every 6 h (q6h), every 3 h (q3h), or every 2 h (q2h)] either standalone or in combination with a zidebactam regimen of total dose of 100 mg/kg, administered in q2h fraction (8.33 mg/kg). MICs of cefepime and cefepime/zidebactam were similar; 2, 1, and 2 mg/L against, *P. aeruginosa* ATCC 27853 (Fig. 4a), PAO1 (Fig. 4b), and S1093 (Fig. 4c), respectively.

Zidebactam monotherapy at total daily dose (TDD) of 100 mg/kg, fractionated q2h showed mortality or no-efficacy. Infrequent regimens of cefepime monotherapy (q24h and q12h) resulted in net-growth or mortality showing lack of effectiveness of such regimens. However, as expected, more frequent regimens (q3h or q2h) resulted in net bacteriostatic effect to ~1 log_10_ kill with cefepime *f*T >MIC being 46.8%–68.2%. Interestingly, q24h and q12h cefepime regimens (ineffective as standalone) combined with zidebactam q2h regimen (ineffective as standalone) turned highly bactericidal (>2 log_10_ kill). At these regimens, cefepime’s *f*T >MIC was just 8%–16% (Fig. 4).

### Morphology of cefepime, zidebactam, or cefepime/zidebactam treated cells

**Fig 5:**
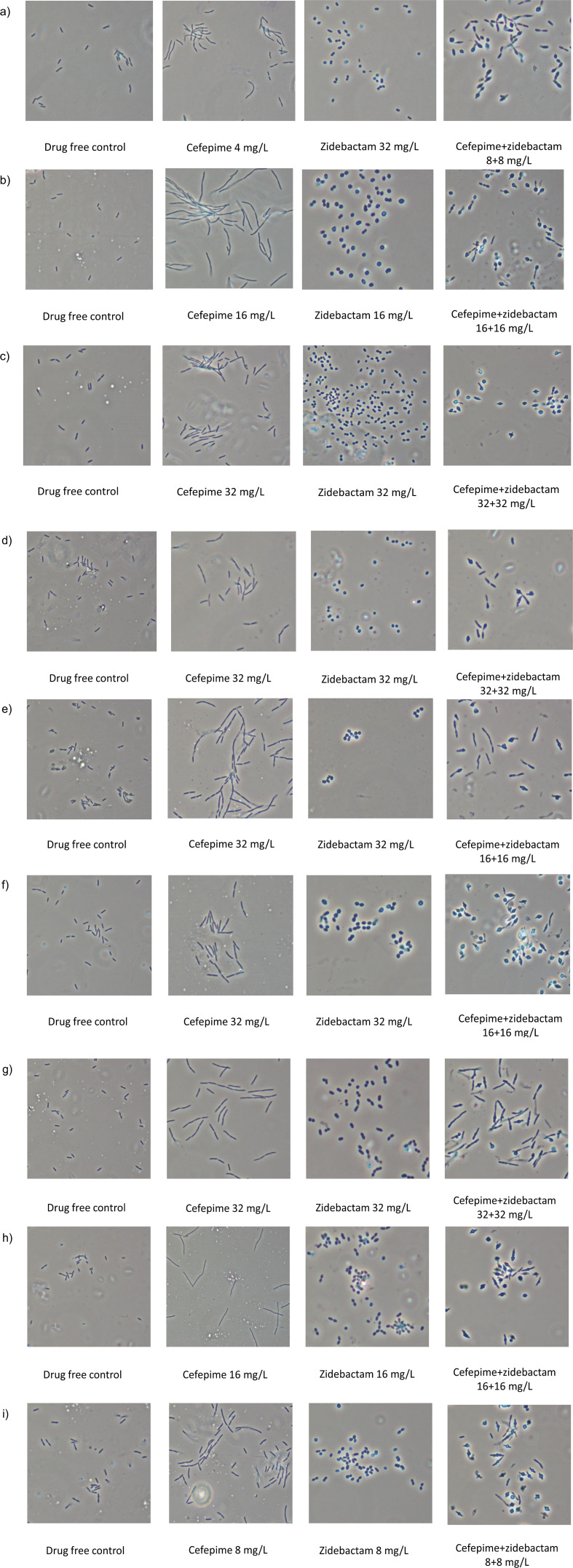
Morphological changes induced by cefepime, zidebactam, and cefepime+zidebactam in *P. aeruginosa* with cefepime/zidebactam MICs 16–32 mg/L (isolates employed in cefepime/zidebactam *in vivo* efficacy study). (**a**) *P. aeruginosa* VA 59; (**b**) *P. aeruginosa* VA 62; (**c**) *P. aeruginosa* VA 88; (**d**) *P. aeruginosa* VA 91; (**e**) *P. aeruginosa* VA 92; (**f**) *P. aeruginosa* VA 93; (**g**) *P. aeruginosa* VA 95; (**h**) *P. aeruginosa* VA 107; and (**i**) *P. aeruginosa* VA 109.

All nine isolates that were treated with zidebactam transformed into spherical forms consistent with an effect of PBP2 inactivation. Cefepime-treated cells showed elongation indicative of PBP3 binding. Cefepime/zidebactam-treated cells were pleomorphic and lysis-prone suggesting bactericidal action associated with synergistic effect of concurrent inactivation of multiple PBPs (Fig. 5).

## DISCUSSION

Contemporary MDR/XDR *P. aeruginosa* phenotypes are characterized by co-expression of various resistance mechanisms, mainly, increased efflux activity, OprD inactivation, PBP mutations and ever evolving β-lactamase variants. The daunting task has been to optimize a single antibiotic that overcomes all these resistance mechanisms. Unfortunately, older as well as newer anti-pseudomonal antibiotics are able to handle only a limited spectrum of resistance mechanisms, thus precluding their use as a reliable monotherapy for contemporary pseudomonal infections. Therefore, for seriously ill patients, combination therapies are required to improve clinical outcomes. However, they pose toxicity risks, adverse PK interactions, and dosing difficulties (29).

Challenged against a set of MDR *P. aeruginosa* isolates possessing diverse resistance mechanisms (PDC, KPC, MBLs, enhanced efflux, OprD inactivation, PBP3 and PBP2 substitutions), the present study revealed the limitations of novel anti-pseudomonal β-lactam/β-lactamase inhibitor combinations. Against this select collection, the susceptibilities to imipenem/relebactam, ceftazidime/avibactam, ceftolozane/tazobactam, and cefepime/taniborbactam were <60% even against a subset of isolates not producing any carbapenemase (overall <50%). This was somewhat unanticipated, since ceftazidime/avibactam, ceftolozane/tazobactam and cefepime/taniborbactam are expected to overcome PDC over-expression (avibactam and taniborbactam being potent β-lactamase inhibitors and ceftolozane being stable to PDC hydrolysis) (30, 31) and OprD truncations (cephalosporins are known to be less impacted than carbapenems) (32, 33). Thus, the modest activities of these β-lactam/β-lactamase inhibitors observed in this study could be attributed to hampered target binding due to the PBP3 substitutions, particularly, in the background of impermeability. Compromise in the activity of imipenem/relebactam could also be attributed to PBP changes, though contribution of concurrently operating additional resistance mechanisms cannot be ruled out.

The probable role of PBP substitutions in impacting the activity of these β-lactam/β-lactamase inhibitors stem from elevated MICs of ceftazidime/avibactam (8 to 32 mg/L) and imipenem/relebactam (16 to >128 mg/L) against ST 1801 isolates harboring KPC-2. Even though both avibactam and relebactam are potent inhibitors of KPC and mutations in genes encoding for hyper efflux were not identified in the ST 1801 isolates, ceftazidime/avibactam and imipenem/relebactam MICs remained high (Table S1). Thus, in conjunction with already well-established resistance mechanisms, proliferation of PBP substitutions in *P. aeruginosa* could further add to the challenge of optimizing novel antibiotics targeted towards this problematic pathogen.

In contrast, β-lactam-enhancer based cefepime/zidebactam demonstrated potent activity against the same set of *P. aeruginosa* isolates with 86.1% susceptibility at a cefepime (2 g, q8h) breakpoint of 8 mg/L which rose to 100% at 32 mg/L, an *in vivo* efficacy-supported cut-off being proposed as cefepime/zidebactam’s PK/PD breakpoint for *P. aeruginosa*. Earlier independent *in vivo* translational studies have established therapeutically relevant coverage of cefepime/zidebactam against *P. aeruginosa* isolates with MICs higher than cefepime’s susceptible breakpoint (up to 32 mg/L) (34, 35).

The consistent *in vitro* activity of cefepime/zidebactam against MDR *P. aeruginosa* isolates expressing a multitude of resistance mechanisms including PBP substitutions is a result of β-lactamase stable zidebactam’s PBP2 binding action that continues even in the isolates expressing enhanced efflux or impermeability as shown in this study. Likewise, cefepime’s ability to engage its high affinity PBP targets is facilitated by rapid cellular penetration and fast rate of PBP binding (14). As a result, in combination, a PBP level synergistic interaction enables cefepime/zidebactam to overcome multiple β-lactam-impacting non-enzymatic and enzymatic resistance mechanisms in *P. aeruginosa*.

Functional evidence of a multiple PBP binding driven synergistic interaction between cefepime and zidebactam was also manifested through changes in morphology of *Pseudomonas* cells. Upon exposure to the cefepime/zidebactam combination, we observed that the cell morphology changed from cocco-bacillary to lysis-prone spheroplasts (indication of multiple PBP engagement). Interestingly, such morphological change was noted at a significantly lower concentration of cefepime when combined with zidebactam, as compared to the concentration of standalone cefepime required to induce elongation (indication of PBP3 binding). Thus, we hypothesize that engagement of PBP3 and PBP2 (multiple target inactivation) by cefepime and zidebactam, respectively, has a synergetic effect by efficiently inhibiting or arresting cell wall synthesis allowing more rapid cell wall degradation (36).

A similar activity profile of cefepime/zidebactam was also proposed by Mullane et al., wherein, the *in vitro* activity of standalone cefepime/zidebactam was compared with several combinations against a panel of 30 carbapenem-resistant *P. aeruginosa* (37). While 97% isolates were inhibited by cefepime/zidebactam alone at ≤16  mg/L, the susceptibility rates to combination of other antibiotics (cefepime, ceftolozane-tazobactam, or meropenem combined with either amikacin or fosfomycin) were lower (<70% at established breakpoints). Pathogen coverage achieved with cefepime/zidebactam alone was even broader than that achieved with the most active combination of ceftolozane/tazobactam plus amikacin or fosfomycin (37).

We further investigated the impact of higher cefepime/zidebactam MICs of 16 and 32 mg/L obtained for nine isolates (Table 3) on its *in vivo* efficacy by employing a neutropenic murine pneumonia model. The results showed that, for all the isolates regardless of MICs, cefepime/zidebactam ELF-HSR caused a ≥ 1-log_10_ kill, thus exceeding the translational end point. Interestingly, for the majority of isolates, even zidebactam monotherapy showed considerable bactericidal effect. Notably, these isolates display several resistance mechanisms such as VIM-2, KPC-2, and PBP3 and PBP2 substitutions. To investigate the PK/PD basis of coverage of *P. aeruginosa* with higher cefepime/zidebactam MIC (higher than cefepime breakpoint of 8 mg/L), we assessed the impact of zidebactam on cefepime’s % *f*T >MIC requirement. This study showed that standalone cefepime *f*T >MIC of ≥46.8% provided merely bacteriostatic to ~1 log_10_ kill effect, while in combination with zidebactam, a lowered cefepime *f*T >MIC of 8%–16% imparted a substantially higher kill of >2 log_10_ CFU. Such modulation of the partner antibiotic’s PK/PD is an attribute associated with zidebactam’s β-lactam enhancer action, not reported with conventional β-lactamase inhibitors. Thus, a lowered requirement of cefepime’s % *f*T >MIC (linked with bactericidal effect) in the presence of zidebactam provided a rationale for observed *in vivo* bactericidal effect of cefepime/zidebactam against *P. aeruginosa* with higher cefepime/zidebactam MICs through its humanized regimen (cefepime 2g + zidebactam 1 g, TID). Moreover, adequacy of shorter % *f*T >MIC requirement of cefepime/zidebactam in rendering bactericidal effect is expected to be beneficial in critically-ill patient population often associated with reduced drug exposures (17).

The *in vivo* efficacy results obtained in this study are in agreement with the Kidd *et al*. study that showed a pronounced *in vivo* efficacy (static to 2 log_10_ kill) of cefepime/zidebactam ELF-HSR against several carbapenem and ceftolozane/tazobactam-resistant *P. aeruginosa* including isolates with cefepime/zidebactam MICs up to 32 mg/L (34). Taking into account the MIC_90_ of cefepime/zidebactam against global isolates (4 mg/L, *n* = 4808) and MIC_90_ against the subset of meropenem-non-susceptible isolates (8 mg/L, *n* = 1147) (38), a translational efficacy of cefepime/zidebactam against *P. aeruginosa* isolates with MICs up to 32 mg/L, as demonstrated in this study, potentially suggests a near-total coverage of MDR/XDR *P. aeruginosa*. If 108 *P. aeruginosa* clinical isolates included in this study were to represent pathogens causing infections in high-resistance regions or in ICU setting, cefepime/zidebactam is expected to be an important future arsenal for the treatment of MDR *P. aeruginosa* infections.

In summary, in the present study, β-lactam enhancer based approach showed promise in overcoming MDR *P. aeruginosa* regardless of resistance mechanisms expressed. While a multitude of resistance mechanisms expressed by MDR *P. aeruginosa* pose severe impediments to newer anti-pseudomonal drugs, the novel β-lactam enhancer approach, as exhibited by zidebactam, shows potential to transcend this challenge.

## MATERIALS AND METHODS

### Bacterial isolates

A collection of 108 well-characterized clinical *P. aeruginosa* isolates was used in this study. These isolates have been collected from northeast Ohio and the Mid-Atlantic states and were stored in the investigator’s laboratory. They were previously determined by phenotypic testing to be carbapenem resistant (>90% of isolates), and most were previously described (39–42).

### Antibiotics and minimum inhibitory concentrations

Zidebactam, avibactam, relebactam, vaborbactam, taniborbactam and ceftolozane were synthesized at Wockhardt Research Centre, Aurangabad, India (>90% HPLC purity). Commercial formulation or >90% pure active pharmaceutical ingredient was used for tazobactam, cefepime, imipenem, meropenem, ciprofloxacin, amikacin, and colistin.

MICs of antibiotics were determined by broth microdilution method as recommended by Clinical & Laboratory Standards Institute, M100 guideline (43). Cefepime/zidebactam MICs were determined at 1:1 ratio. The fixed inhibitor concentration of 4 mg/L was used for avibactam, relebactam, tazobactam, and taniborbactam while 8 mg/L was used for vaborbactam. FDA breakpoints were employed for determining the susceptibility rates of isolates to the comparator antibiotics.

### Whole genome sequencing (WGS)

WGS of all the study isolates (*n* = 108) was performed. Briefly, the sequencing libraries were prepared using the Nextra DNA Flex library preparation kit (Illumina, San Diego, CA) as per the manufacturer’s instructions. Subsequently, the paired-end library was subjected to sequencing on a HiSeq 2500 platform (Illumina, USA) generating 2 × 150 bp reads. Sequencing reads with a PHRED quality score below 20 were discarded and adapters were trimmed using cutadapt v1.8.1 and assessed with FastQC v0.11.4. Draft genome sequence data generated using Illumina were assembled using SPAdes (v.3.13.0) (44). Genome assemblies were annotated using the Prokaryotic Genome Annotation Pipeline (PGAP v.4.1) from NCBI (45).

Core genome single-nucleotide polymorphisms (SNPs) were identified using Snippy v.0.2.6 (https://github.com/tseemann/snippy) with *P. aeruginosa* PAO1 (accession no. CP053028.1) as the reference.

### *In vivo* efficacy

#### Assessment of cefepime/zidebactam efficacy employing ELF-HSR

*In vivo* efficacy employing HSR of cefepime alone, or zidebactam alone, or cefepime/zidebactam was evaluated in a murine neutropenic infection model as described previously (34) against isolates with cefepime/zidebactam MICs of >8 mg/L (Table 3). These dosing regimens (designed at Wockhardt) produced cefepime, or zidebactam, or cefepime plus zidebactam exposures in mice epithelial lining fluid (ELF) comparable to that of respective exposures obtained in human ELF after 2 + 1 g, q8h administration of cefepime/zidebactam. The comparability between mice and human exposures in the ELF was in terms of the proportion of time during which cefepime and zidebactam concentrations remained above cefepime/zidebactam MICs (Table S2A and B; Fig. S1). Male/female Swiss Albino mice were rendered neutropenic by intra-peritoneal injections of cyclophosphamide 150 and 100 mg/kg on 4 days and 1 day prior to the infection, respectively. The humanized regimen of cefepime/zidebactam in mice was rendered feasible by slowing the renal elimination with the help of uranyl nitrate 5 mg/kg, intra-peritoneal injection administered 3 days before the infection (46).

Animals were infected with 0.05 mL of normal saline containing ~10^7^ CFU/ml of *P. aeruginosa* through nostrils under isofluorane-induced transient anesthesia. Nine *P. aeruginosa* with cefepime/zidebactam MICs of 16–32 mg/L with resistance to imipenem/relebactam and ceftolozane/tazobactam were employed in this study (Table 3). Treatment (subcutaneous injections) was initiated 2 h post-infection with cefepime HSR or zidebactam HSR or cefepime/zidebactam combination HSR. A group of animals was administered with vehicle control. After 24-h treatment duration, animals were humanely sacrificed, and the lung bacterial load was estimated. Earlier at the time of initiation of treatment (0 h), a group of infected mice was sacrificed to determine the lung bacterial burden at time 0 h. Efficacy was defined as change in the bacterial load at 24 h as compared to 0 h. All the groups consisted of six animals.

### Effect on cefepime’s % *f*T>MIC requirement in the presence of zidebactam

The above-described murine neutropenic infection model was employed with the exception that animals were not administered with uranyl nitrate. Cefepime was administered as a single dose (q24h) or in fractionated regimens over 24 h; every 12 h (q12h), every 6 h (q6h), every 3 h (q3h), or every 2 h (q2h) and the same regimen was combined with zidebactam given 8.33 mg/kg, every 2 h (q2h). The infecting isolates were cefepime-susceptible (*n* = 3) which enabled determining the efficacy-linked magnitude of % *f*T >MIC for cefepime monotherapy, which could then be compared with that of cefepime in the presence of zidebactam. The % *f*T >MIC of cefepime in the various fractionated regimens was determined using non-linear sigmoidal E_max_ model (GraphPad Prism version 7) and previously reported mouse plasma PK of cefepime (18).

### Morphology of cefepime, zidebactam, or cefepime/zidebactam-treated cells

Morphological changes were studied for isolates with cefepime/zidebactam MICs of 16–32 mg/L by exposing sub-minimum inhibitory concentrations or at inhibitory concentrations of zidebactam or cefepime or cefepime/zidebactam to 10^6^ CFU/mL bacterial density in cation-adjusted Mueller-Hinton broth under shaking condition. The treated cells were visualized after 3 h of exposure using a phase contrast microscope.

## Data Availability

Genome assemblies were submitted to the National Center for Biotechnology Information (NCBI) GenBank database (BioProject no. PRJNA869185).

## AFTER EPUB

*[This article was published on 27 October 2023 with text missing from the Introduction, 4th paragraph. The Introduction was corrected in the current version, posted on 1 November 2023.]*

## SUPPLEMENTAL MATERIAL

Supplemental Tables and Figures mbio.01118-23-s0001.docx

ASM does not own the copyrights to Supplemental Material that may be linked to, or accessed through, an article. The authors have granted ASM a non-exclusive, world-wide license to publish the Supplemental Material files. Please contact the corresponding author directly for reuse.
