# Supplementary material for: Transcending the challenge of evolving resistance mechanisms in Pseudomonas aeruginosa through β-lactam-enhancer-mechanism-based cefepime/zidebactam
Source: mBio. 2023 Oct 27;14(6):e01118-23. doi: 10.1128/mbio.01118-23 (PMC10746216; doi:10.1128/mbio.01118-23)
Supplement: Supplemental Tables and Figures [file mbio.01118-23-s0001.docx]

| **Isolates & MLST** | **MICs (mg/L)** | **β-lactamases** | **OprD** | **AmpR** | **MexB** | **MexR** | **NalC** | **PBP2** | **PBP3** |
| --- | --- | --- | --- | --- | --- | --- | --- | --- | --- |
| VA02 (ST 235) | IMP: >128  MEM: 128  I/R: >128  C/A: 32  T/T: >128  5222: 2 | OXA-488, PDC-35,  VIM-2 | *T103S, K115T, F170L, E185Q, P186G, V189T, R310E, A315G, G425A | G283E, M288R | WT | -1M, -2N,  -3Y, -4P, M5V, V126E | G71E, E153Q, S209R | WT | WT |
| VA03 (ST 233) | IMP: >128  MEM: 128  I/R: >128  C/A: 64  T/T: >128  5222: 2 | OXA-4,  OXA-486, PDC-3,  VIM-2 | deletion results in FS and stop at 144 | WT | Stop at 712 | -1M, -2N,  -3Y, -4P, M5V | G71E, E153D, A186T | WT | WT |
| VA04 (ST 179) | IMP: 8  MEM: 2  I/R: 2  C/A: 2  T/T: 1  5222: 4 | OXA-396, PDC-8 | Stop at 50 | WT | WT | -1M, -2N,  -3Y, -4P, M5V | G71E, S209R | WT | A530T |
| VA05 (ST 308) | IMP: >128  MEM: >128  I/R: >128  C/A: >128  T/T: >128  5222: 8 | NDM-1,  OXA-488, PDC-19a | T1-IV | G283E, M288R | WT | -1M, -2N,  -3Y, -4P, M5V, V126E | G71E, D79E, S209R | WT | WT |
| VA06 (ST 111) | IMP: >128  MEM: 64  I/R: >128  C/A: 32  T/T: 128  5222: 4 | OXA-395, PDC-3,  VIM-2 | Stop at 277 | G283E, E287G, M288Q, A290V, V291L, A293S, R294E, G295A, R296- | WT | -1M, -2N,  -3Y, -4P, M5V, V126E | G71E | WT | WT |
| VA08 (ST 356) | IMP: 8  MEM: 4  I/R: 8  C/A: 32  T/T: 2  5222: 16 | OXA-50, PDC-3 | T1-VI | WT | G461S | -1M, -2N,  -3Y, -4P, M5V, I72V | M1-, N2-, D3-, A4-, S5-, P6-, R7M, T9L, E10P, R11G, G71E | WT | WT |
| VA09 (ST 446) | IMP: 16  MEM: 16  I/R: 2  C/A: 64  T/T: 64  5222: 4 | OXA-226, OXA-848, PDC-16 | *R310E, A315G, G425A, T103S, K115T, F170L, E185Q, P186G, V189T | R86C, E114A, G283E, M288R | WT | -1M, -2N,  -3Y, -4P, M5V, V126E | G71E, A145V, S209R | WT | WT |
| VA10 (ST 2100) | IMP: 4  MEM: 64  I/R: 2  C/A: >128  T/T: >128  5222: 32 | OXA-486, PDC-537 | stop at 93 | G283E, E287G, M288Q, A290V, V291L, A293S, R294E, G295A, R296- | P565S, E818G | -1M, -2N,  -3Y, -4P, M5V, V126E | G71E, S209R | G591S | G63D, R504C |
| VA11 (ST 654) | IMP: 32  MEM: 64  I/R: 8  C/A: 64  T/T: 64  5222: 8 | OXA-396, PDC-3,  PME-1 | *-26F, V127L, E185Q, P186G, V189T, E202Q, I210A, E230K, S240T, N262T, T276A, A281G, K296Q, Q301E, R310E, G312R, A315G, L347M, M372V, S373-, N375S, N376S, V377S, G378S, K380A, N381G, Y382L, G383-, S407A, Q428E | WT | WT | -1M, -2N,  -3Y, -4P, M5V | G71E | WT | F533L |
| VA12 (ST 446) | IMP: 32  MEM: 128  I/R: 2  C/A: 64  T/T: 64  5222: 8 | OXA-226, OXA-848, PDC-16 | *T103S, K115T, F170L, E185Q, P186G, V189T, R301E, A315G, G245A | R86C, E114A, G283E, M288R | WT | -1M, -2N,  -3Y, -4P, M5V, V126E | G71E, A145V, S209R | WT | F533L |
| VA14 (ST 308) | IMP: 1  MEM: 0.5  I/R: 0.25  C/A: 2  T/T: 0.25  5222: 2 | OXA-488, PDC-19a | TI-IV | G283E, M288R | WT | -1M, -2N,  -3Y, -4P, M5V, V126E | G71E, D79E, S209R | WT | WT |
| VA15 (ST 463) | IMP: 16  MEM: 4  I/R: 2  C/A: 2  T/T: 4  5222: 2 | OXA-486, PDC-8 | stop at 163 | WT | I747V | -1M, -2N,  -3Y, -4P, M5V | G71E, S209R | WT | WT |
| VA16 (ST 2100) | IMP: 2  MEM: 4  I/R: 2  C/A: 128  T/T: 16  5222: 4 | OXA-486, PDC-479 | stop at 94 | G283E, E287G, M288Q, A290V, V291L, A293S, R294E, G295A, R296- | DEL 1-202, V203M, P326L | -1M, -2N,  -3Y, -4P, M5V, V126E | G71E, S209R | WT | A244T |
| VA17 (ST 446) | IMP: 1  MEM: 2  I/R: 0.25  C/A: 16  T/T: 1  5222: 8 | OXA-2,  OXA-848, PDC-16 | TI-IV | E114A, G283E, M288R | WT | -1M, -2N,  -3Y, -4P, M5V, V126E | G71E, A145V, S209R | V232G | A60E |
| VA18 (ST 274) | IMP: 32  MEM: 32  I/R: 4  C/A: 8  T/T: 8  5222: 4 | OXA-486, PDC-24 | stop at 417 | WT | WT | -1M, -2N,  -3Y, -4P, M5V, S88-, D89-, Q90-, R91- | G71E, S209R | WT | WT |
| VA20 (ST 274) | IMP: 32  MEM: 32  I/R: 4  C/A: 8  T/T: 0.5  5222: 8 | OXA-486, PDC-24 | stop at 417 | WT | WT | -1M, -2N,  -3Y, -4P, M5V, S88-, D89-, Q90-, R91- | G71E, S209R | WT | WT |
| VA21 (ST 2100) | IMP: 4  MEM: 8  I/R: 2  C/A: >128  T/T: >128  5222: 4 | OXA-486, PDC-537 | Stop at 93 | G283E, E287G, M288Q, A290V, V291L, A293S, R294E, G295A, R296- | P565S | -1M, -2N,  -3Y, -4P, M5V, V126E | G71E, S209R | WT | G63D, R504C |
| VA22 (ST 27) | IMP: 64  MEM: 16  I/R: 8  C/A: 64  T/T: 64  5222: 4 | OXA-494, PDC-15 | stop at 347 | WT | WT | -1M, -2N,  -3Y, -4P, M5V, V126E, V132A | G71E, S209R | WT | WT |
| VA23 (ST 27) | IMP: 32  MEM: 32  I/R: 8  C/A: 64  T/T: 4  5222: 8 | OXA-494, PDC-15 | stop at 277 | WT | WT | -1M, -2N,  -3Y, -4P, M5V, V126E, L131P, V132A | G71E, S209R | WT | WT |
| VA24 (ST 175) | IMP: 16  MEM: 0.5  I/R: 1  C/A: 1  T/T: 0.5  5222: 2 | OXA-50, PDC-1 | T1-VI | WT | WT | -1M, -2N,  -3Y, -4P, M5V | G71E, A186T | WT | P527S |
| VA25 (ST 1596) | IMP: 16  MEM: 8  I/R: 1  C/A: 128  T/T: 8  5222: 8 | OXA-50, PDC-3 | T1-VI | D135N | WT | -1M, -2N,  -3Y, -4P, M5V | G71E, S209R | WT | WT |
| VA26 (ST 2100) | IMP: 4  MEM: 64  I/R: 4  C/A: >128  T/T: >128  5222: 16 | OXA-486,  PDC-537 | stop at 94 | G283E, E287G, M288Q, A290V, V291L, A293S, R294E, G295A, R296- | P565S | -1M, -2N,  -3Y, -4P, M5V, V126E | G71E, S209R | WT | G63D, R504C |
| VA27 (ST 2100) | IMP: 4  MEM: 64  I/R: 2  C/A: >128  T/T: >128  5222: 32 | OXA-486,  PDC-537 | stop at 94 | G283E, E287G, M288Q, A290V, V291L, A293S, R294E, G295A, R296- | P565S | -1M, -2N,  -3Y, -4P, M5V, V126E | G71E, S209R | G591S | G63D, R504C |
| VA28 (ST 2100) | IMP: 0.12  MEM: 2  I/R: 0.12  C/A: >128  T/T: >128  5222: 2 | OXA-486,  PDC-537 | stop at 94 | G283E, E287G, M288Q, A290V, V291L, A293S, R294E, G295A, R296- | P565S | -1M, -2N,  -3Y, -4P, M5V, V126E | G71E, S209R | WT | G63D, R504C |
| VA29 (ST 2100) | IMP: 0.12  MEM: 4  I/R: 0.12  C/A: >128  T/T: >128  5222: 4 | OXA-486,  PDC-537 | stop at 94 | G283E, E287G, M288Q, A290V, V291L, A293S, R294E, G295A, R296- | DEL 1-202, V203M, P565S | -1M, -2N,  -3Y, -4P, M5V, V126E | G71E, S209R | WT | G63D, R504C |
| VA30 (ST 2100) | IMP: 1  MEM: 16  I/R: 1  C/A: >128  T/T: >128  5222: 8 | OXA-486,  PDC-537 | stop at 94 | G283E, E287G, M288Q, A290V, V291L, A293S, R294E, G295A, R296- | P565S | -1M, -2N,  -3Y, -4P, M5V, V126E | G71E, S209R | WT | G63D, R504C |
| VA31 (ST 463) | IMP: 16  MEM: 4  I/R: 2  C/A: 2  T/T: 0.5  5222: 2 | OXA-486, PDC-8 | stop at 163 | E114A, G283E, M288R | WT | -1M, -2N,  -3Y, -4P, M5V, V126E | G71E, A145V, S209R | WT | WT |
| VA32 (ST 298) | IMP: 8  MEM: 8  I/R: 1  C/A: 16  T/T: 8  5222: 8 | OXA-848, PDC-16 | *stop at 54 | WT | WT | -1M, -2N,  -3Y, -4P, M5V, R63H, V126E, L131P, V132A | G71E, S209R | Not found | WT |
| VA33 (ST 27) | IMP: 32  MEM: 32  I/R: 4  C/A: 128  T/T: 64  5222: 8 | OXA-494, PDC-15 | stop at 277 | G283E, E287G, M288Q, A290V, V291L, A293S, R294E, G295A, R296- | WT | -1M, -2N,  -3Y, -4P, M5V, V126E | G71E, S209R | WT | A244T |
| VA34 (ST 2100) | IMP: 4  MEM: 4  I/R: 2  C/A: 8  T/T: 8  5222: 2 | OXA-486, PDC-382 | stop at 277 | G283E, M288R | WT | -1M, -2N,  -3Y, -4P, M5V, V126E | G71E, E153Q, S209R | WT | WT |
| VA35 (ST 235) | IMP: 32  MEM: 32  I/R: 8  C/A: 128  T/T: 16  5222: 8 | OXA-1184, OXA-488, PDC-35 | *T103S, K115T, F170L, E185Q, P186G, V189T, R310E, A315G, G425A | E114A, G283E, M288R | WT | -1M, -2N, -3Y, -4P, M5V, V126E | G71E, A145V, S209R | WT | WT |
| VA36 (ST 2100) | IMP: 4  MEM: 32  I/R: 4  C/A: >128  T/T: >128  5222: 16 | OXA-486, PDC-537 | stop at 94 | G283E, E287G, M288Q, A290V, V291L, A293S, R294E, G295A, R296- | P565S | -1M, -2N,  -3Y, -4P, M5V, V126E | G71E, S209R | WT | G63D, R504C |
| VA37 (ST 298) | IMP: 1  MEM: 16  I/R: 1  C/A: >128  T/T: >128  5222: 8 | OXA-848, PDC-16 | *stop at 54 | E114A, G283E, M288R | WT | -1M, -2N,  -3Y, -4P, M5V, V126E | G71E, A145V, S209R | WT | WT |
| VA38 (ST 2100) | IMP: 4  MEM: 32  I/R: 4  C/A: >128  T/T: >128  5222: 8 | OXA-486, PDC-537 | stop at 54 | G283E, E287G, M288Q, A290V, V291L, A293S, R294E, G295A, R296- | P565S | -1M, -2N,  -3Y, -4P, M5V, V126E | G71E, S209R | WT | G63D, R504C |
| VA39 (ST 2100) | IMP: 8  MEM: 128  I/R: 8  C/A: >128  T/T: >128  5222: 32 | OXA-486, PDC-537 | stop at 54 | G283E, E287G, M288Q, A290V, V291L, A293S, R294E, G295A, R296- | P565S | -1M, -2N,  -3Y, -4P, M5V, V126E | G71E, S209R | G591S | G63D, R504C |
| VA40 (ST 1435) | IMP: 32  MEM: 32  I/R: 8  C/A: 64  T/T: 2  5222: 8 | OXA-486, PDC-302 | del 1-8 | G283E, M288R | WT | Del 1-19, V20M, V126E | G71E | WT | WT |
| VA41 (ST 298) | IMP: 32  MEM: 16  I/R: 4  C/A: 16  T/T: 2  5222: 8 | OXA-848, PDC-16 | * Stop at 94 | E114A, G283E, M288R | WT | -1M, -2N,  -3Y, -4P, M5V, V126E | G71E, A145V, S209R | WT | WT |
| VA42 (ST 179) | IMP: 16  MEM: 32  I/R: 4  C/A: 8  T/T: 1  5222: 4 | OXA-396, PDC-8 | stop at 50 | WT | WT | -1M, -2N,  -3Y, -4P, M5V | G71E, S209R | WT | A530T |
| VA43 (ST 298) | IMP: 32  MEM: 16  I/R: 4  C/A: 16  T/T: 1  5222: 8 | OXA-848, PDC-16 | * Stop at 94 | E114A, G283E, M288R | WT | -1M, -2N,  -3Y, -4P, M5V, V126E | G71E, A145V, S209R | WT | WT |
| VA44 | IMP: 16  MEM: 8  I/R: 1  C/A: 1  T/T: 0.5  5222: 1 | OXA-1035, PDC-19a | missing 1-277 | G283E, M288R | WT | -1M, -2N,  -3Y, -4P, M5V, V126E | G71E, S209R | WT | N117S |
| VA45 (ST 571) | IMP: 32  MEM: 32  I/R: 4  C/A: 4  T/T: 0.5  5222: 4 | OXA-494, PDC-218 | stop at 417 | G283E, M288R | WT | Del 5-19,  V20M, R21C | G71E, S209R | WT | WT |
| VA46 (new ST) | IMP: 32  MEM: 4  I/R: 1  C/A: 8  T/T: >128  5222: 2 | OXA-488, PDC-35 | stop at 238 | G283E, M288R | WT | -1M, -2N,  -3Y, -4P, M5V, V126E | G71E, E153Q, S209R | WT | WT |
| VA47 (ST 253) | IMP: 32  MEM: 16  I/R: 4  C/A: 8  T/T: 1  5222: 4 | OXA-488, PDC-34 | stop at 42 | E114A, G283E, M288R | WT | -1M, -2N,  -3Y, -4P, M5V, V126E | G71E, A145V, S209R | WT | WT |
| VA48 (ST 298) | IMP: 32  MEM: 32  I/R: 8  C/A: 64  T/T: 2  5222: 8 | OXA-848, PDC-16 | *stop at 94 | E114A, G283E, M288R | WT | -1M, -2N,  -3Y, -4P, M5V, V126E | G71E, A145V, S209R | WT | WT |
| VA49 (ST 362) | IMP: >128  MEM: 32  I/R: 2  C/A: 8  T/T: 64  5222: 8 | KPC-2,  OXA-904, PDC-3 | TI-VII (except S57E, S59R, D437E) | WT | WT | -1M, -2N,  -3Y, -4P, M5V | G71E, S209R | WT | WT |
| VA50 (ST 111) | IMP: 64  MEM: 8  I/R: 64  C/A: 32  T/T: 128  5222: 8 | OXA-395, PDC-3,  VIM-2 | TI-VII (except S57E, S59R, D437E) | G283E, E287G, M288, A290V, V291L, A293S, R294E, G295A, R296- | WT | -1M, -2N,  -3Y, -4P, M5V, V126E | G71E | WT | WT |
| VA51 (ST 298) | IMP: 16  MEM: 4  I/R: 1  C/A: 1  T/T: 0.5  5222: 2 | OXA-848, PDC-16 | *stop at 54 | E114A, G283E, M288R | WT | -1M, -2N,  -3Y, -4P, M5V, V126E | G71E, A145V, S209R | WT | WT |
| VA52 (ST 179) | IMP: 32  MEM: 64  I/R: 2  C/A: 8  T/T: 2  5222: 8 | OXA-396, PDC-8 | stop at 51 | WT | WT | -1M, -2N,  -3Y, -4P, M5V | G71E, S209R | WT | A530T |
| VA53 (ST 298) | IMP: 16  MEM: 16  I/R: 4  C/A: 4  T/T: 0.5  5222: 2 | OXA-848, PDC-16 | * K114T, T103S,  K170L,  E185Q, P186G, V189T, R310E, A315G, G425A | E114A, G283E, M288R | WT | -1M, -2N,  -3Y, -4P, M5V, L95P, V126E | G71E, A145V, S209R | WT | WT |
| VA54 (ST 308) | IMP: 64  MEM: 16  I/R: 32  C/A: 32  T/T: 128  5222: 2 | OXA-488, PDC-19a, VIM-2 | T1-IV | G283E, M288R | WT | -1M, -2N,  -3Y, -4P, M5V, V126E | G71E, D79E, S209R | WT | WT |
| VA55 (ST 571) | IMP: 32  MEM: 16  I/R: 2  C/A: 8  T/T: 0.5  5222: 4 | OXA-494, PDC-218 | stop at 417 | G283E, M288R | WT | M5-, N6-, P7-, D8-, L9-, M10-, P11-,  A12-, L13-, M14-, A15-,  V16-,  F17-,  Q18-, H19-, V20M, R21C | G71E, S209R | WT | WT |
| VA56 (ST 27) | IMP: 64  MEM: 16  I/R: 4  C/A: 8  T/T: 4  5222: 8 | OXA-494, PDC-15 | stop at 277 | D135N | WT | -1M, -2N,  -3Y, -4P, M5V, V126E, V132A | G71E, S209R | WT | WT |
| VA57 (ST 1249) | IMP: 64  MEM: 64  I/R: 64  C/A: 64  T/T: >128  5222: 4 | OXA-2,  OXA-908, PDC-115, VIM-2 | T1-IV | G283E, M288R | I186V | -1M, -2N,  -3Y, -4P, M5V, V126E | G71E, A145V, S209R | WT | A139S |
| VA58 (ST 532) | IMP: 128  MEM: 32  I/R: 64  C/A: 128  T/T: >128  5222: 4 | OXA-906, PDC-59,  VIM-2 | T1-IV | E114A, G283E, M288R | WT | -1M, -2N,  -3Y, -4P, M5V, V126E | G71E, A145V, S209R | WT | WT |
| VA59 (ST 233) | IMP: >128  MEM: 128  I/R: >128  C/A: 128  T/T: >128  5222: 32 | OXA-4,  OXA-486,  PDC-3,  VIM-2 | T1-VI | WT | E81V | -1M, -2N,  -3Y, -4P, M5V | G71E, E153D, A186T | WT | WT |
| VA60 (ST 235) | IMP: 32  MEM: 8  I/R: 4  C/A: 2  T/T: 1  5222: 2 | OXA-2,  OXA-488, PDC-35 | missing normal stop at 476 (frame shift at 402) +32 AA | G283E, M288R | WT | -1M, -2N,  -3Y, -4P, M5V, V126E | G71E, E153Q, S209R | WT | WT |
| VA61 (ST 1284) | IMP: 32  MEM: 16  I/R: 2  C/A: 4  T/T: 0.5  5222: 4 | OXA-488, PDC-39 | *stop at 349 | E114A, G283E, M288R | WT | -1M, -2N,  -3Y, -4P, M5V, A110T, V126E, T130P | G71E, A145V, S209R | WT | WT |
| VA62 (ST 1801) | IMP: >128  MEM: >128  I/R: 64  C/A: 16  T/T: 64  5222: 16 | OXA-10, OXA-486, PDC-3 | stop at 202 | WT | WT | -1M, -2N,  -3Y, -4P, M5V | G71E, S209R | A174V, V517M | F533L |
| VA63 (ST 235) | IMP: >128  MEM: >128  I/R: 8  C/A: 64  T/T: 64  5222: 4 | KPC-2,  OXA-1183,  OXA-488, PDC-35 | missing normal stop at 443 (frame shift at 440) +29 AA | G283E, M288R | WT | -1M, -2N,  -3Y, -4P, M5V, V126E | G71E, E153Q, S209R | WT | WT |
| VA64 (ST 235) | IMP: 32  MEM: 32  I/R: 2  C/A: 16  T/T: 8  5222: 8 | OXA-488, PDC-16 | stop at 202 | G283E, M288R | WT | -1M, -2N,  -3Y, -4P, M5V, V126E | G71E, E153Q, S209R | WT | WT |
| VA65 (ST 298) | IMP: 16  MEM: 4  I/R: 2  C/A: 1  T/T: 0.5  5222: 4 | OXA-848, PDC-16 | *LL insert at 46, T103S, K115T, F170L, E185Q, P186G, V189T, R310E, A315G, G425A | E114A, G283E, M288R | WT | -1M, -2N,  -3Y, -4P, M5V, V126E | G71E, A145V, S209R | WT | WT |
| VA66 (ST 389) | IMP: 32  MEM: 32  I/R: 4  C/A: 16  T/T: 4  5222: 8 | OXA-50, PDC-1 | stop at 359 | WT | -638V,  -639P | -1M, -2N,  -3Y, -4P, M5V | G71E, S209R | WT | WT |
| VA67 (ST 446) | IMP: 32  MEM: 8  I/R: 1  C/A: 32  T/T: 64  5222: 2 | OXA-2,  OXA-848,  PDC-16 | *T103S, K115T, F170L, E185Q, P186G, V189T, R310E, A315G, G425A | E114A,  G283E,  M288R | WT | V126E | G71E, A145V, S209R | WT | WT |
| VA68 (ST 233) | IMP: 64  MEM: 8  I/R: 64  C/A: >128  T/T: >128  5222: 8 | OXA-4,  OXA-486, PDC-3,  VIM-2 | T1-VI | WT | E81V | -1M, -2N,  -3Y, -4P, M5V | G71E, E153D, A186T | WT | WT |
| VA69 (ST 571) | IMP: 32  MEM: 32  I/R: 4  C/A: 16  T/T: 2  5222: 4 | OXA-494, PDC-218 | stop at 417 | G283E, M288R | WT | del 1-19, V20M, R21C | G71E, S209R | WT | WT |
| VA70 (ST 282) | IMP: >128  MEM: >128  I/R: 8  C/A: 1  T/T: 16  5222: 4 | KPC-2,  OXA-50, PDC-103 | stop at 324* IS4-like transposase | WT | WT | -1M, -2N,  -3Y, -4P, M5V | G71E, S209R, P210L | WT | T91A |
| VA71 (ST 571) | IMP: 32  MEM: 16  I/R: 2  C/A: 8  T/T: 0.5  5222: 4 | OXA-494, PDC-218 | stop at 417 | G283E, M288R | WT | del 1-19, V20M, R21C | G71E, S209R | WT | WT |
| VA72 (ST 571) | IMP: 32  MEM: 16  I/R: 2  C/A: 4  T/T: 0.5  5222: 2 | OXA-494, PDC-218 | stop at 417 | G283E, M288R | WT | del 1-19, V20M, R21C | G71E, S209R | WT | WT |
| VA73 (new ST) | IMP: 64  MEM: 8  I/R: 2  C/A: 8  T/T: 4  5222: 4 | OXA-50, PDC-3 | stop at 114 | D135N | WT | -1M, -2N,  -3Y, -4P, M5V | G71E, S209R | WT | WT |
| VA74 (ST 179) | IMP: 8  MEM: 16  I/R: 1  C/A: 16  T/T: 4  5222: 4 | OXA-396, PDC-8 | stop at 54 | WT | WT | -1M, -2N,  -3Y, -4P, M5V | G71E, S209R | WT | A530T |
| VA75 (ST 235) | IMP: 16  MEM: >128  I/R: 2  C/A: 32  T/T: 64  5222: 8 | KPC-2,  OXA-1183  OXA-488, PDC-35 | stop at 339 | G283E, M288R | WT | -1M, -2N,  -3Y, -4P, M5V, V126E | G71E, E153Q, S209R | WT | WT |
| VA76 (ST 253) | IMP: 16  MEM: 8  I/R: 1  C/A: 2  T/T: 1  5222: 2 | OXA-488, PDC-34 | del1-31 | E114A, G283E, M288R | WT | -1M, -2N,  -3Y, -4P, M5V, V126E | Not found | WT | WT |
| VA77 (ST 298) | IMP: 16  MEM: 4  I/R: 1  C/A: 1  T/T: 0.25  5222: 4 | OXA-848, PDC-16 | *stop at 95 | E114A, G283E, M288R | WT | -1M, -2N,  -3Y, -4P, M5V, V126E | G71E, A145V, S209R | WT | WT |
| VA78 (ST 253) | IMP: 16  MEM: 16  I/R: 2  C/A: 4  T/T: 2  5222: 8 | OXA-488, PDC-34 | stop at 142 | E114A, G283E, M288R | WT | -1M, -2N,  -3Y, -4P, M5V, V126E | G71E, A145V, S209R | WT | WT |
| VA79 (ST 282) | IMP: >128  MEM: >128  I/R: 32  C/A: 4  T/T: 8  5222: 8 | KPC-2, OXA-50, PDC-103 | NOT Found | WT | WT | -1M, -2N,  -3Y, -4P, M5V | T36P, G71E, S209R, P210L | WT | T91A |
| VA80 (ST 571) | IMP: 32  MEM: 32  I/R: 2  C/A: 4  T/T: 0.5  5222: 4 | OXA-494, PDC-218 | stop at 417 | G283E, M288R | WT | del 1-19, V20M, R21C | G71E, S209R | WT | WT |
| VA81 (-) | IMP: 128  MEM: 32  I/R: 1  C/A: 2  T/T: 32  5222: 8 | KPC-2, OXA-1030, PDC-5 | T1-VI | WT | WT | -1M, -2N,  -3Y, -4P, M5V, Q60H | G71E, S209R | WT | WT |
| VA82 (ST 253) | IMP: 16  MEM: 64  I/R: 2  C/A: 8  T/T: 2  5222: 8 | OXA-488, PDC-34 | del 1-30 | E114A, G283E, M288R | WT | -1M, -2N,  -3Y, -4P, M5V, L95H, V126E | G71E, A145V, S209R | WT | WT |
| VA83 (ST 571) | IMP: 32  MEM: 16  I/R: 2  C/A: 4  T/T: 0.5  5222: 4 | OXA-494 | stop at 417 | Not found | WT | del 1-19, V20M, R21C | G71E, S209R | WT | WT |
| VA84 (ST 111) | IMP: 16  MEM: 2  I/R: 0.5  C/A: 0.5  T/T: 0.12  5222: 0.5 | OXA-395, PDC-1 | stop at 425 | WT | WT | -1M, -2N,  -3Y, -4P, M5V | G71E, A186T | WT | WT |
| VA85 (ST 1803) | IMP: >128  MEM: >128  I/R: 32  C/A: 4  T/T: 32  5222: 8 | KPC-2, OXA-10, OXA-395, PDC-36 | deletion 1-111 | E114A, G283E, M288R | WT | -1M, -2N,  -3Y, -4P, M5V,  A12-, L13-, M14-, A15-, V126E | G71E, E153Q, S209R | WT | WT |
| VA86 (ST 282) | IMP: 128  MEM: >128  I/R: 16  C/A: 8  T/T: 64  5222: 8 | KPC-5, OXA-2, OXA-50, PDC-103 | Not found | WT | WT | -1M, -2N,  -3Y, -4P, M5V | T36P, G71E, S209R, P210L | WT | T91A |
| VA87 (ST 244) | IMP: >128  MEM: >128  I/R: 16  C/A: 16  T/T: 128  5222: 8 | KPC-2, OXA-847, PDC-1 | frame shift, loss of normal stop +71 AA | WT | WT | -1M, -2N,  -3Y, -4P, M5V | WT | WT | WT |
| VA88 (ST 1801) | IMP: 128  MEM: >128  I/R: 128  C/A: 16  T/T: 128  5222: 32 | KPC-2, OXA-10, OXA-486, PDC-3 | stop at 202 | WT | WT | -1M, -2N,  -3Y, -4P, M5V | G71E, S209R | A174V, V517M | F533L |
| VA89 (ST 282) | IMP: >128  MEM: >128  I/R: 4  C/A: 2  T/T: 8  5222: 4 | KPC-2, OXA-50, PDC-103 | Not found | WT | WT | -1M, -2N,  -3Y, -4P, M5V | T36P, G71E, S209R, P210L | WT | T91A |
| VA90 (ST 233) | IMP: >128  MEM: >128  I/R: 16  C/A: 8  T/T: 64  5222: 8 | KPC-2, OXA-10, OXA-486, PDC-3 | stop at 202 | WT | WT | -1M, -2N,  -3Y, -4P, M5V | G71E, S209R | A174V, V517M | F533L |
| VA91 (ST 1801) | IMP: >128  MEM: >128  I/R: 128  C/A: 8  T/T: 128  5222: 32 | KPC-2, OXA-10, OXA-486, PDC-3 | stop at 202 | WT | WT | -1M, -2N,  -3Y, -4P, M5V | G71E, S209R | A174V, V517M | F533L |
| VA92 (ST 1801) | IMP: >128  MEM: >128  I/R: 128  C/A: 32  T/T: 128  5222: 16 | KPC-2, OXA-10, OXA-486, PDC-3 | stop at 202 | WT | WT | -1M, -2N,  -3Y, -4P, M5V | G71E, S209R | A174V, V517M | F533L |
| VA93 (ST 1801) | IMP: >128  MEM: >128  I/R: 64  C/A: 8  T/T: 128  5222: 16 | KPC-2, OXA-10, OXA-486, PDC-3 | stop at 202 | WT | WT | -1M, -2N,  -3Y, -4P, M5V | G71E, S209R | A174V, V517M | F533L |
| VA94 (ST 282) | IMP: >128  MEM: >128  I/R: 8  C/A: 2  T/T: 32  5222: 4 | KPC-2, OXA-2, OXA-50, PDC-103 | stop at 324*  IS4-like transposase | WT | WT | -1M, -2N,  -3Y, -4P, M5V | G71E, S209R, P210L | WT | T91A |
| VA95 (ST 1801) | IMP: >128  MEM: >128  I/R: >128  C/A: 16  T/T: 128  5222: 32 | KPC-2, OXA-10, OXA-486, PDC-3 | stop at 202 | WT | WT | -1M, -2N,  -3Y, -4P, M5V | G71E, S209R | A174V, V517M | F533L |
| VA96 (ST 362) | IMP: >128  MEM: 16  I/R: 0.5  C/A: 8  T/T: 32  5222: 4 | KPC-2, OXA-904, PDC-3 | TI-VII (except S57E, S59R, D437E) | WT | WT | -1M, -2N,  -3Y, -4P, M5V | G71E, S209R | WT | WT |
| VA97 (ST 282) | IMP: >128  MEM: >128  I/R: 4  C/A: 2  T/T: 8  5222: 4 | KPC-2, OXA-50, PDC-103 | Not found | WT | WT | -1M, -2N,  -3Y, -4P, M5V | T36P, G71E, S209R, P210L | WT | T91A |
| VA98 (ST 233) | IMP: 128  MEM: 16  I/R: 128  C/A: 64  T/T: >128  5222: 4 | OXA-4, OXA-486, PDC-3, VIM-2 | TI-VI | WT | WT | -1M, -2N,  -3Y, -4P, M5V | G71E, E153D, A186T | WT | WT |
| VA100 (ST 446) | IMP: 16  MEM: 8  I/R: 1  C/A: 2  T/T: 0.25  5222: 2 | OXA-848, PDC-16 | stop at 346 | E114A, G283E, M288R | WT | -1M, -2N,  -3Y, -4P, M5V, L95V, V126E | G71E, A145V, S209R | WT | WT |
| VA101 (ST 245) | IMP: 32  MEM: 16  I/R: 4  C/A: 16  T/T: 4  5222: 8 | OXA-494, PDC-5 | stop at 306 | WT | WT | -1M, -2N,  -3Y, -4P, M5V | G71E, S209R | WT | WT |
| VA102 (ST 232) | IMP: 32  MEM: 32  I/R: 4  C/A: 8  T/T: 1  5222: 4 | OXA-396, PDC-5 | stop at 215 | R244W, G283E, E287G, M288Q, A290V, V291L, A293S, R294E, G295A, R296Q, + 10AA | WT | -1M, -2N,  -3Y, -4P, M5V,  S88-,  D89-, Q90-, R91- | G71E, S209R, P210L | WT | WT |
| VA103 (ST 389) | IMP: 32  MEM: 16  I/R: 2  C/A: 4  T/T: 1  5222: 8 | OXA-50, PDC-1 | stop at 277 | WT | WT | -1M, -2N,  -3Y, -4P, M5V | G71E, S209R | WT | WT |
| VA104 (ST 233) | IMP: 128  MEM: 64  I/R: 128  C/A: 64  T/T: >128  5222: 4 | OXA-4, OXA-486, PDC-3, VIM-2 | T1-VI | WT | WT | -1M, -2N,  -3Y, -4P, M5V | G71E, E153D, A186T | WT | WT |
| VA105 (ST 233) | IMP: 128  MEM: 64  I/R: 128  C/A: 128  T/T: >128  5222: 8 | OXA-4, OXA-486, PDC-3, VIM-2 | stop at 226 | WT | WT | -1M, -2N,  -3Y, -4P, M5V | G71E, E153D, A186T | WT | WT |
| VA106 (ST 233) | IMP: >128  MEM: 128  I/R: >128  C/A: 64  T/T: >128  5222: 4 | OXA-4, OXA-486, PDC-3, VIM-2 | stop at 226 | WT | WT | -1M, -2N,  -3Y, -4P, M5V | G71E, E153D, A186T | WT | WT |
| VA107 (ST 233) | IMP: >128  MEM: 128  I/R: >128  C/A: 128  T/T: >128  5222: 16 | OXA-4, OXA-486, PDC-3, VIM-2 | T1-VI | WT | WT | del 1-61 R78K, N79K, L80P, V81G, R82P, R83P, E84R, R85A | G71E, E153D, A186T | WT | WT |
| VA108 (ST 233) | IMP: >128  MEM: 64  I/R: 128  C/A: 128  T/T: >128  5222: 4 | OXA-4, OXA-486, PDC-3, VIM-2 | T1-VI | WT | WT | -1M, -2N,  -3Y, -4P, M5V | G71E, E153D, A186T | WT | WT |
| VA109 (ST 233) | IMP: >128  MEM: 128  I/R: >128  C/A: 128  T/T: >128  5222: 16 | OXA-4, OXA-486, PDC-3, VIM-2 | T1-VI | WT | E81V | -1M, -2N, -3Y, -4P, M5V | G71E, E153D, A186T | WT | WT |
| VA110 (ST 2100) | IMP: 2  MEM: 64  I/R: 2  C/A: 16  T/T: 16  5222: 4 | OXA-486, PDC-481 | stop at 194 | G283E, E287G, M288Q, A290V, V291L, A293S, R294E, G295A, R296- | WT | -1M, -2N,  -3Y, -4P, M5V, V126E | G71E, S209R | WT | A244T |
| VA111 (ST 2100) | IMP: 0.5  MEM: 8  I/R: 0.5  C/A: >128  T/T: 128  5222: 4 | OXA-486, PDC-479 | stop at 94 | G283E, E287G, M288Q, A290V, V291L, A293S, R294E, G295A, R296- | S180L, Stop 652 | -1M, -2N,  -3Y, -4P, M5V, V126E | G71E, S209R | WT | A244T |
| VA112 (ST 233) | IMP: 1  MEM: 16  I/R: 1  C/A: >128  T/T: >128  5222: 8 | OXA-4, OXA-486,  PDC-3, VIM-2 | T1-VI | WT | WT | -1M, -2N,  -3Y, -4P, M5V | G71E, E153D, A186T | WT | WT |
| VA113 (ST 2100) | IMP: 4  MEM: 8  I/R: 2  C/A: 8  T/T: 32  5222: 8 | OXA-486, PDC-481 | stop at 94 | G283E, E287G, M288Q, A290V, V291L, A293S, R294E, G295A, R296- | V107M | -1M, -2N,  -3Y, -4P, M5V, V126E | G71E, S209R | WT | A244T |

**Supplementary Table 1. Antibiotic resistance determinants identified from whole genome sequence.** *Denotes on two contigs.

WT = protein sequence that is the same as the reference isolate (*P. aeruginosa* PAO1; OprD types based on PMID 22290967.

| Time (h)* | Mouse doses (mg/kg body weight) | |
| --- | --- | --- |
|  | Cefepime | Zidebactam |
| 0 | 40 | 20 |
| 2 | 10 | 5 |
| 4 | 5 | 2 |
| 6 | 4 | 2 |

**Supplementary Table 2A.** Human epithelial lining fluid (ELF)-simulated dose regimens of cefepime and zidebactam employed in the neutropenic mouse lung infection model. *Doses were repeated every 8 h for a 24 h period. These doses generated cefepime and zidebactam exposures in mouse ELF that were equivalent to human ELF exposures obtained with cefepime/zidebactam dose regimen of 2 g + 1 g, q8h, 60-min infusion.

| **Drug regimen** | **Species** | **% T>concentration** | | | | | | | |
| --- | --- | --- | --- | --- | --- | --- | --- | --- | --- |
|  |  | **1** | **2** | **4** | **8** | **16** | **32** | **64** | **128** |
| **Cefepime 2 g, q8h** | **Human** | 99.8 | 99.6 | 95.6 | 70.7 | 45.0 | 10.7 | 0.0 | 0.0 |
|  | **Mouse** | 99.9 | 99.8 | 99.6 | 54.9 | 35.7 | 7.2 | 0.0 | 0.0 |
|  |  |  |  |  |  |  |  |  |  |
| **Zidebactam 1 g, q8h** | **Human** | 99.6 | 88.9 | 63.1 | 36.3 | 0.0 | 0.0 | 0.0 | 0.0 |
|  | **Mouse** | 99.8 | 86.4 | 52.6 | 35.2 | 2.8 | 0.0 | 0.0 | 0.0 |

**Supplementary Table 2B.** Comparison of %T>cefepime or zidebactam concentrations in the human ELF (administered with cefepime/zidebactam 2 + 1 g, q8h, 60-min infusion) and in mouse ELF (administered with human ELF-simulated dose regimen).

**a) Cefepime time-concentration profile**

**
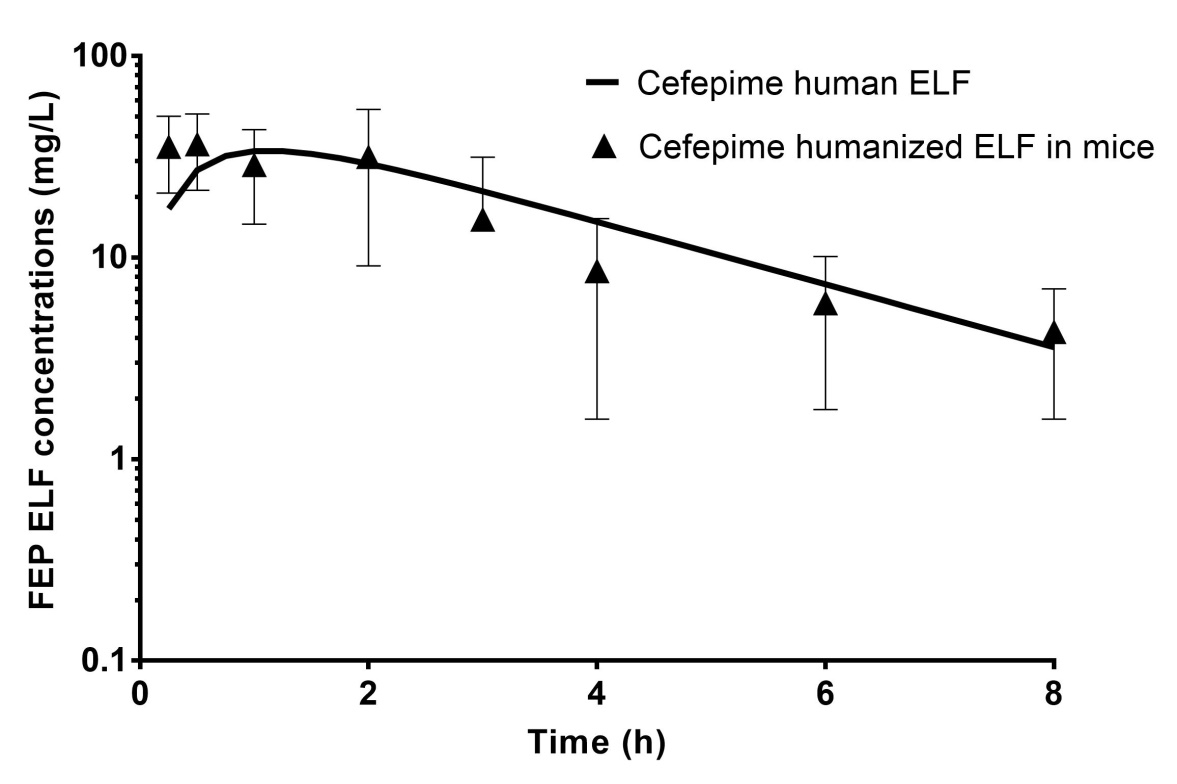
**

**b) Zidebactam time-concentration profile**


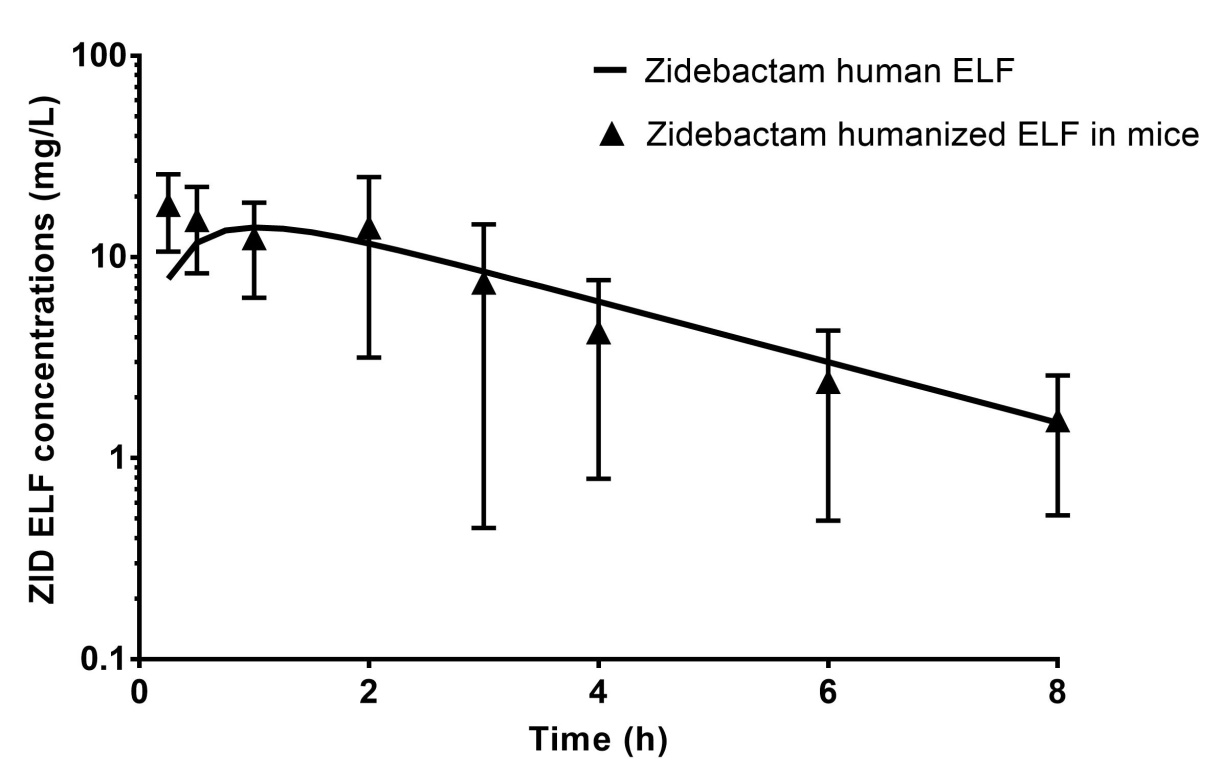


**Supplementary Figure S1.** Time-concentration profile of cefepime (a) and zidebactam (b) in ELF obtained with clinical regimen and human ELF-simulated regimen (ELF-HSR). The human time-concentration profile of cefepime and zidebactam was from cefepime/zidebactam Phase 1 studies. For mice time-concentration profile, mean of data from five mice was used for each estimate.

8 isolates were inhibited by FEP/ZID 16 to 32 mg/L for which in vivo efficacy of FEP/ZID was demonstrated

29 isolates inhibited by FEP/ZID ≤ 8 mg/L

**Supplementary Figure S2. Distribution of MICs of cefepime/zidebactam versus other β-lactam/ β-lactamase inhibitor combinations for each carbapenemase-producing isolate (n = 37).** FEP/ZID: cefepime/zidebactam, FEP/TAN: cefepime/taniborbactam, CAZ/AVI: ceftazidime/avibactam, TOL/TAZ: ceftolozane/tazobactam, IPM/REL: imipenem/relebactam.
